# Molecular Subsets in the Gene Expression Signatures of Scleroderma Skin

**DOI:** 10.1371/journal.pone.0002696

**Published:** 2008-07-16

**Authors:** Ausra Milano, Sarah A. Pendergrass, Jennifer L. Sargent, Lacy K. George, Timothy H. McCalmont, M. Kari Connolly, Michael L. Whitfield

**Affiliations:** 1 Department of Genetics, Dartmouth Medical School, Hanover, New Hampshire, United States of America; 2 Norris Cotton Cancer Center, Dartmouth Medical School, Hanover, New Hampshire, United States of America; 3 Department of Dermatology, University of California San Francisco, San Francisco, California, United States of America; 4 Department of Medicine (Rheumatology), University of California San Francisco, San Francisco, California, United States of America; 5 Department of Pathology, University of California San Francisco, San Francisco, California, United States of America; University College Dublin, Ireland

## Abstract

**Background:**

Scleroderma is a clinically heterogeneous disease with a complex phenotype. The disease is characterized by vascular dysfunction, tissue fibrosis, internal organ dysfunction, and immune dysfunction resulting in autoantibody production.

**Methodology and Findings:**

We analyzed the genome-wide patterns of gene expression with DNA microarrays in skin biopsies from distinct scleroderma subsets including 17 patients with systemic sclerosis (SSc) with diffuse scleroderma (dSSc), 7 patients with SSc with limited scleroderma (lSSc), 3 patients with morphea, and 6 healthy controls. 61 skin biopsies were analyzed in a total of 75 microarray hybridizations. Analysis by hierarchical clustering demonstrates nearly identical patterns of gene expression in 17 out of 22 of the forearm and back skin pairs of SSc patients. Using this property of the gene expression, we selected a set of ‘intrinsic’ genes and analyzed the inherent data-driven groupings. Distinct patterns of gene expression separate patients with dSSc from those with lSSc and both are easily distinguished from normal controls. Our data show three distinct patient groups among the patients with dSSc and two groups among patients with lSSc. Each group can be distinguished by unique gene expression signatures indicative of proliferating cells, immune infiltrates and a fibrotic program. The intrinsic groups are statistically significant (p<0.001) and each has been mapped to clinical covariates of modified Rodnan skin score, interstitial lung disease, gastrointestinal involvement, digital ulcers, Raynaud's phenomenon and disease duration. We report a 177-gene signature that is associated with severity of skin disease in dSSc.

**Conclusions and Significance:**

Genome-wide gene expression profiling of skin biopsies demonstrates that the heterogeneity in scleroderma can be measured quantitatively with DNA microarrays. The diversity in gene expression demonstrates multiple distinct gene expression programs in the skin of patients with scleroderma.

## Introduction

Scleroderma is a systemic autoimmune disease with a heterogeneous and complex phenotype that encompasses several distinct subtypes. The disease has an estimated prevalence of 276 cases per million adults in the United States [1], [2]. Median age of onset is 45 years of age with the ratio of females to males being approximately 4∶1.

Scleroderma is divided into distinct clinical subsets. One subset is the localized form, which affects skin only including morphea, linear scleroderma and eosinophilic fasciitis. The other major type is systemic sclerosis (SSc) and its subsets. The most widely recognized classification system for SSc divides patients into two subtypes, diffuse and limited, a distinction made primarily by the degree of skin involvement [3]. Patients with SSc with diffuse scleroderma (dSSc) have severe skin involvement [4] often characterized by more rapid onset and progressive course with fibrotic skin involvement extending from the hands and arms, trunk, face and lower extremities. Patients with SSc with limited scleroderma (lSSc) have fibrotic skin involvement that is typically limited to the fingers (sclerodactyly), hands and face. Some patients in the limited subset develop significant pulmonary arterial hypertension, pulmonary fibrosis or digital ischemia/ulcerations. Although there are certain disease characteristics that differentiate these two groups, some of the severe vascular and organ manifestations occur across groups and are the cause of significant morbidity and mortality [5].

Disease classification based largely on the extent of skin involvement does not reflect the true heterogeneity of scleroderma [6], [7]. We have used a genomic approach to capture the clinical diversity among different patients in a way that will provide new insight into the complexity of the disease. High throughput gene expression data, combined with clinical phenotypic data, provides a powerful new tool to probe the underlying biology of scleroderma.

Skin thickening is one of the earliest manifestations of the disease; it remains the most sensitive and specific finding [8] and is one of the most widely used outcome measures in clinical trials [9], [10], [11]. Several studies have demonstrated that the extent of skin involvement directly correlates with internal organ involvement and prognosis in SSc patients [12], [13], [14]. Furthermore, improvement in Modified Rodnan Skin Score (MRSS) is associated with improved survival [15]. Probing the gene expression in this end target organ is likely to yield genes that will provide clues to pathogenesis and may serve as potential biomarkers of disease activity. In this study we have measured the genome-wide patterns of gene expression in skin biopsies from patients with SSc because skin can provide insights into the relevant pathological processes in the disease.

DNA microarrays have been used to characterize the changes in gene expression that occur in dSSc skin when compared to normal controls [16], [17]. Here we extend these findings to show that DNA microarrays can measure the heterogeneity in scleroderma skin. We identify molecular subsets among dSSc and define a gene expression signature that is associated with lSSc. We also identify a subgroup that contains skin biopsies from patients with dSSc, lSSc and localized scleroderma (morphea), characterized by a unified gene expression signature indicative of an early inflammatory response. Each gene expression subgroup has been mapped to clinical covariates and biological processes that are modified in the disease.

## Results

Previous studies have demonstrated that the skin of patients with dSSc can be easily distinguished from normal controls at the level of gene expression [16], [17]. Here, we have extended these findings and tested the hypothesis that we can identify distinct subsets of scleroderma within the existing clinical classifications by gene expression profiling of skin biopsies using DNA microarrays.

We studied skin biopsies from 34 subjects: twenty-four patients with SSc (17 dSSc and 7 lSSc), 3 patients with morphea and 6 healthy controls (**Tables 1** **–** **2**). A single biopsy was analyzed from a patient with eosinophilic fasciitis (EF). Skin biopsies were taken from two different anatomical sites for 27 subjects: a forearm site, and a lower back site. In dSSc, the forearm site was clinically affected and the back site was clinically unaffected. In lSSc, both forearm and back sites were clinically unaffected. Seven subjects provided single biopsies resulting in a total of 61 biopsies. Total RNA was prepared from each skin biopsy and analyzed on whole-genome DNA microarrays. In addition, fourteen technical replicates were analyzed for a total of 75 microarray hybridizations.

**Table 1:** Subject clinical characteristics.

Subject	Age/Sex	Duration, yrs	Skin Score (0–51)	Raynaud's severity (0–10)	Digital Ulcers (0–3)	GI	ILD	Renal	ANA/Scl-70/ACA
dSSc 1	41/F	2	28	-	0	+	+	−	+/+/−
dSSc 2	49/M	2.5	26	3	0	+	−	−	ND
dSSc 3	33/F	2.5	35	7	0	−	−	−	+/+/−
dSSc 4	47/F	3	35	7	0	+	−	−	+/−/−
dSSc 5	52/F	1	10	4	1	+	−	−	+/+/−
dSSc 6	63/F	0.5	26	10	0	−	−	−	+/−/−
dSSc 7	42/F	2.5	23	10	3	+	−	−	ND
dSSc 8	58/M	2	43	7	0	−	−	−	+/−/−
dSSc 9	56/F	8	21	5	0	+	+	−	+/−/−
dSSc 10	35/F	7	35	8	2	+	+	−	−/−/−
dSSc 11	47/F	8.5	30	8	1	+	+	−	+/+/−
dSSc 12	58/M	9	15	5	0	+	−	−	−/−/−
dSSc 13	47/F	6	15	3	0	+	−	−	+/−/−
dSSc 14	49/F	10	15	8	0	−	+	−	+/−/−
dSSc 15	58/F	20	18	2	1	+	+	−	ND
dSSc 16	65/F	10	20	4	0	+	+	+	ND
dSSc 17	40/F	20	15	2	1	+	+	+	ND
lSSc 1	67/F	3	8	5	0	+	−	−	+/−/+
lSSc 2	57/F	2	8	2	0	+	−	−	+/−/+
lSSc 3	35/F	3	6	6	3	+	−	−	+/−/−
lSSc 4	63/F	13	8	6	0	−	+	−	+/−/−
lSSc 5	60/F	28	9	6	0	+	+	+	+/−/−
lSSc 6	55/F	17	9	6	1	+	+	−	+/−/−
lSSc 7	67/F	5	8	5	0	+	+	−	+/+/−

Clinical characteristics of the 25 Systemic Sclerosis subjects from which skin biopsies were taken are shown. Indicated for each subject are the age, sex, disease duration since first onset of non-Raynaud's symptoms, modified Rodnan skin score on a 51-point scale, a self-reported Raynaud's severity score on a 10-point scale, and the presence or absence of digital ulcers on a 3-point scale. Also indicated are the presence (+) or absence (−) of gastrointestinal involvement (GI), interstitial lung disease (ILD) as determined by high-resolution computerized tomography (HRCT), and renal disease. The age and sex of subjects with Morphea are: Morph1 (49 yrs, female, disease duration 16 yrs), Morph2 (54 yrs, female, disease duration 7 yrs), and Morph3 (49 yrs, female, disease duration 4 yrs). The age and sex of healthy control subjects are as follows: Nor1, 53 yrs, female; Nor2, 47 yrs, female; Nor3, 41, female; Nor4, 26, female; Nor5, 45, male; Nor6, 29, female. ND = Not determined

**Table 2:** Skin samples collected and microarrays hybridized.

Diagnosis	Patients	Biopsies	Microarrays
Diffuse SSc	17	30	38
Limited SSc	7	14	16
Morphea	3	4	5
Normal	6	12	15
Eosinophilic fasciitis	1	1	1
**Total**	**34**	**61**	**75**

### Overview of the gene expression profiles

**Figure 1:**
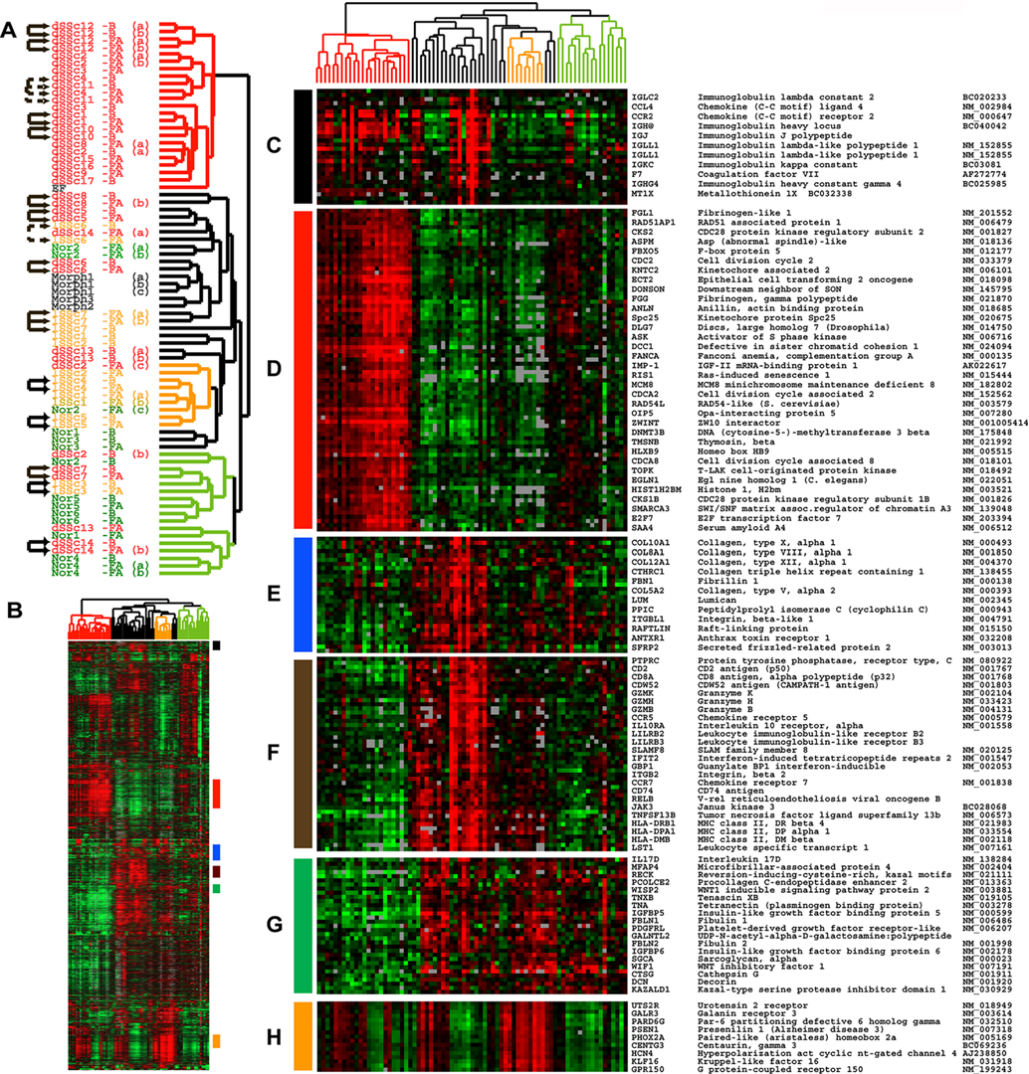
Gene expression signatures in scleroderma. 4,149 probes that changed at least 2-fold from their median value on at least two microarrays were selected from 75 microarray hybridizations representing 61 biopsies. Probes and microarrays were ordered by 2-dimensional average linkage hierarchical clustering. This clustering shows that the dSSc, lSSc, morphea samples form distinct groups largely stratified by their clinical diagnosis. A. The unsupervised hierarchical clustering dendrogram shows the relationship among the samples using this list of 4,149 probes. Samples names have been color-coded by their clinical diagnosis: dSSc in red, lSSc in orange, morphea and EF in black, and healthy controls (Nor) in green. Forearm (FA) and Back (B) are indicated for each sample. Solid arrows indicate the 14 of 22 forearm-back pairs that cluster next to one another; dashed arrows indicate the additional 3 forearm-back pairs that cluster with only a single sample between them. Technical replicates are indicated by the labels (a), (b) or (c). 9 out of 14 technical replicates cluster immediately beside one another. B. Overview of the gene expression profiles for the 4,149 probes. Each probe has been centered on its median expression value across all samples analyzed. Measurements that are above the median are colored red and those below the median are colored green. The intensity of the color is directly proportional to the fold change. Groups of genes on the right hand side indicated with colored bars are shown in greater detail in panels C–H. C. Immunoglobulin genes expressed highly in a subset of patients with dSSc and in patients with morphea, D. proliferation signature, E. collagen and extracelluar matrix components, F. genes typically associated with the presence of T-lymphocyes and macrophages, G. Genes showing low expression in dSSc, H. Heterogeneous expression cluster that is high in lSSc and a subset of dSSc. In each case only a subset of the genes in each cluster are shown. The precise location of each gene in the cluster can be viewed in Supplemental Figure S1.

We identified 4,149 probes whose expression varied from their median values in these samples by more than 2-fold in at least two of the 75 arrays and analyzed them by two-dimensional hierarchical clustering [18]. The resulting sample dendrogram shows that the samples separate into two main branches (**Figure 1A**) that in part stratify patients by their clinical diagnosis. The branch lengths in the tree are inversely proportional to the correlation between samples or groups of samples. The diversity in gene expression among the patients with scleroderma is greater than previously shown (**Figure 1B**) [16], [17] as distinct subsets of scleroderma are evident in the gene expression patterns. Some of these delineate existing classifications, such as the distinction between limited and diffuse, while others reflect new groups. One subset of dSSc patients cluster on the left branch (red) and has gene expression profiles that are distinct from both healthy controls and patients with lSSc (**Figure 1B–1C**), while a second subset of dSSc skin clusters in the middle of the dendrogram tree (black), and a third set clusters with healthy controls. We found lSSc samples formed a group in the middle portion of the dendrogram and could be associated with a distinct, but heterogeneous gene expression signature that also showed high expression in a subset of dSSc patients (**Figure 1H**). LSSc samples are partially intermixed with normal controls on the right boundary and with dSSc on the left boundary of the tree, illustrating that their gene expression phenotype is highly variable (**Figure 1A**). Samples taken from individuals with morphea also grouped together with a gene expression signatures that overlapped with those of dSSc and lSSc (**Figure 1**). Although nodes can be flipped, we have left the nodes of the dendrogram as originally organized by the clustering software, which places nodes with the most similar samples next to one another. Although, the assignment of samples into particular clusters (**Table 3**) would not change even if nodes were flipped.

**Table 3:** Cluster assignments using the scleroderma intrinsic genes.

Patient	Cluster 3.0	Sig Cluster	Consensus Cluster Assignment		
Identifier	Assignment	(p<0.001)	K = 4	K = 5	K = 6
dSSc2 *	Diffuse 1	1	[1 or 3]	[1 or 5]	[1 or 5]
dSSc12	Diffuse 1	1	1	1	1
dSSc1	Diffuse 2	1	1	1	1
dSSc10	Diffuse 2	1	1	1	1
dSSc11	Diffuse 2	1	1	1	1
dSSc15	Diffuse 2	1	1	1	1
dSSc16	Diffuse 2	1	1	1	1
dSSc17	Diffuse 2	1	1	1	1
dSSc3	Diffuse 2	1	1	1	1
dSSc4	Diffuse 2	1	1	1	1
dSSc9	Diffuse 2	1	1	1	1
dSSc8 *	Inflammatory	[5]	2	2	2
dSSc5	Inflammatory	2	2	2	2
dSSc6	Inflammatory	2	2	2	2
lSSc6	Inflammatory	2	2	2	2
lSSc7	Inflammatory	2	2	2	2
Morph1	Inflammatory	2	2	2	2
Morph2	Inflammatory	2	2	2	2
Morph3	Inflammatory	2	2	2	2
lSSc1	Limited	4	4	4	4
lSSc4	Limited	4	4	4	4
lSSc5	Limited	4	4	4	4
Nor1	Limited	4	4	4	4
lSSc2	Normal-like	3	4	4	4
Nor2	Normal-like	3	4	4	4
Nor3	Normal-like	3	4	4	4
dSSc14	Normal-like	3	3	3	3
dSSc7	Normal-like	3	3	3	3
lSSc3	Normal-like	3	3	3	3
Nor4	Normal-like	3	3	3	3
Nor5	Normal-like	3	3	3	3
Nor6	Normal-like	3	3	3	3
dSSc13 *	Unclassified	1	[4]	[4]	[4]
EF *	Unclassified	1	1	1	[6]

* Inconsistently classified

Multiple distinct gene expression programs are evident in each subgroup. Some of these recapitulate the major themes in our prior microarray study of dSSc skin [16] while others reflect gene expression programs not previously observed. A subset of these biological themes and selected genes are discussed below. The entire figure with all gene names is available in the supplementary material (**Supplementary Figure S1; Supplementary Data File S1**).

Immunoglobulins typically associated with B lymphocytes and plasma cells are expressed in a subset of the dSSc skin biopsies (**Figure 1C**). Previously we found dense clusters of infiltrating B cells in dSSc by immunohistochemistry (IHC), indicating that these genes may be from infiltrating CD20+ B cells rather than from a small number of infiltrating plasma cells [16].

Previous studies have identified infiltrating T cells in the skin of dSSc patients [19], [20], [21], [22], [23], although an association between T cell gene expression and dSSc was not observed in our prior study [16]. A new result from this study is genes typically associated with T cells are more highly expressed in a subset of the patients (**Figure 1F**). These genes include the PTPRC (CD45; Leukocyte Common Antigen Precursor), which is required for T-cell activation through the antigen receptor [24], [25], [26], as well as CD2 [27], [28] and CDW52 [29] that are expressed on the surface of T lymphocytes. Also found were CD8A, Granzyme K, Granzyme H, and Granzyme B that are typically expressed in cytotoxic T lymphocytes [30], [31], [32], [33], [34], and CCR7, which is expressed in B and T lymphocytes [35]. Genes induced by interferon (IFIT2, GBP1), genes involved in antigen presentation (HLA-DRB1, HLA-DPA1 and HLA-DMB) and CD74, the receptor for Macrophage Inhibitory factor (MIF), are also present [36], [37], [38], [39], [40]. Genes typically associated with the monocyte/macrophage lineage, B cells and dendritic cells (DCs) were also found in this cluster including Leukocyte immunoglobulin-like receptor B2 and B3 (LILRB2 and LILRB3; **Figure 1F**) [41], [42]. Finally, genes specific to the monocyte/macrophage lineage such as CD163 are expressed in this group of genes (data not shown) [43].

Genes typically associated with the process of fibrosis were co-expressed with markers of T lymphocytes and macrophages. These genes showed increased expression in the central group of samples that included patients with dSSc, lSSc and morphea (**Figure 1E**). Included in this set of genes were the collagens (COL5A2, COL8A1, COL10A1, COL12A1), and collagen triple helix repeat containing 1 (CTHRC1), which is typically expressed in vascular calcifications of diseased arteries and has been shown to inhibit TGF−β signaling [44], [45] (**Figure 1E**). Also found in this cluster was fibrillin-1 (FBN1). The phenotype of the TSK1 mouse, a model of scleroderma, results from a partial in-frame duplication of the FBN1 gene and defects in FBN1 are the cause of Marfan's syndrome (OMIM: 154700).

A surprising result in this study is the differential expression of a ‘proliferation signature’ (**Figure 1D**). The proliferation signature is defined as genes that are expressed only when cells are dividing [46]. We have previously shown that the proliferation signature, originally identified in breast cancer [47], [48], is composed almost completely of cell cycle-regulated genes [49]. Genes showing increased expression in this cluster include the cell cycle-regulated genes CKS1B, CDKS2, CDC2, MCM8, and E2F7 [49]. The existence of a proliferation signature is consistent with reports demonstrating that a subset of cells in dSSc skin biopsies show high levels of tritiated thymidine uptake indicative of cells undergoing DNA replication [50], [51]; a more recent study has shown increased expression of the cell cycle-regulated gene PCNA in a perivascular distribution [52]. IHC of dSSc skin biopsies with the proliferation marker KI67 also shows proliferating cells primarily in the epidermis (see below).

Another cluster of genes is expressed at low levels in the dSSc skin biopsies but at higher levels in all other biopsies, however it is not clearly associated with a single biological function or process. Included in this cluster are the genes WIF1, Tetranectin, IGFBP6, and IGFBP5 found in our original study [16] with similar patterns of expression (**Figure 1G**).

Since the skin of lSSc patients does not show any clinical or histologic manifestations at the biopsy site, it was possible that the skin of those patients would not show significant differences in gene expression when compared to normal controls. In fact, lSSc skin showed a distinct, disease-specific gene expression profile. This novel finding demonstrates that microarrays are sensitive enough to identify the limited subset of SSc even when discernable skin fibrosis was not present. There is a signature of genes that is expressed at high levels in a subset of lSSc patients, and variably expressed in dSSc and normal controls (**Figure 1H**). Included in this signature is the urotensin 2 receptor (UTS2R). The ligand for this receptor, urotensin 2, is considered to be one of the most potent vasoconstrictors yet identified [53], [54], [55]. This finding raises the intriguing possibility that this vasoactive peptide is involved in the vascular pathogenesis of lSSc.

We previously demonstrated that skin biopsies from patients with early dSSc show nearly identical patterns of gene expression at a clinically affected forearm site and a clinically unaffected back site, and the gene expression profiles are distinct from those found in healthy controls [16]. This finding is confirmed in this larger cohort of patients analyzed on a different microarray platform. 14 of 22 forearm-back pairs cluster immediately next to one another indicating that these samples are more similar to each other than to any other sample (**Figure 1A**). An additional 3 forearm-back pairs grouped together with only a single sample between them (**Figure 1A**). In total, 17 of 22 (77%) forearm-back pairs show nearly identical patterns of gene expression. This result holds true for patients with lSSc even though neither the forearm or back biopsy sites in lSSc patients are defined as clinically affected [16]. We found 9 out of 14 technical replicates clustered next to one another. The five technical replicates that did not cluster together are likely misclassified as a result of noise in the genes selected by fold change.

### Classification of scleroderma using the intrinsic genes from skin biopsies

**Figure 2:**
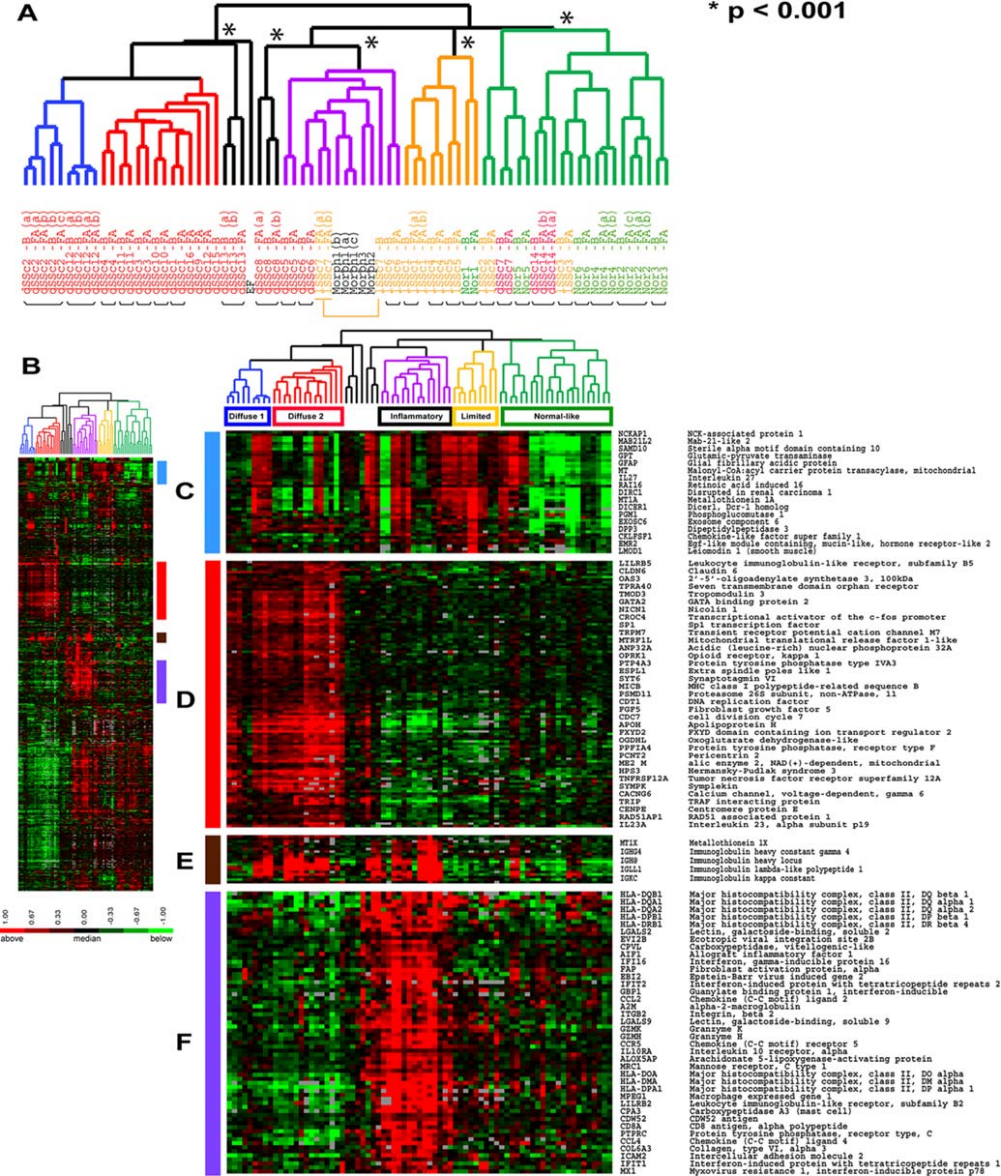
Cluster analysis using the scleroderma intrinsic gene set. The 995 most ‘intrinsic’ genes selected from 75 microarray hybridizations analyzing 34 individuals. Two major branches of the dendrogram tree are evident which divide a subset of the dSSc samples from all other samples. Within these major groups are smaller branches with identifiable biological themes, which have been colored accordingly: blue for *diffuse 1*, red for *diffuse 2*, purple for *inflammatory*, orange for *limited* and green for *normal-like*. Statistically significant clusters (p<0.001) identified by SigClust are indicated by an asterisk (*) at the lowest significant branch. A. Experimental sample hierarchical clustering dendrogram. Black bars indicate forearm-back pairs which cluster together based on this analysis. B. Scaled down overview of the intrinsic gene expression signatures. C. Limited SSc gene expression cluster. D. Proliferation cluster. E. Immunoglobulin gene expression cluster. F. T-lymphocyte and IFNγ gene expression cluster. The full figure with all gene names can be viewed in Supplemental Figure S2.

A list of genes selected by their fold change alone is not ideal for classifying samples because they emphasize differences between samples rather than the intrinsic differences between patients [47], [56]. To select genes that captured the intrinsic differences between patients, we exploited the observation that the forearm-back pairs from each SSc patient show nearly identical patterns of gene expression to select the ‘intrinsic’ genes in SSc. We selected 995 genes with the most consistent expression between each forearm-back pair and technical replicates, but with the highest variance across all samples analyzed [47], [56] (**Supplementary Data File S2**). Each of the 995 intrinsic genes was centered on its median value across all experiments, and the data clustered hierarchically in both the gene and experiment dimension using average linkage hierarchical clustering. The dendrogram summarizes the relationship among the samples and shows their clear separation into distinct groups (**Figure 2A**). As a direct result of our gene selection, all forearm-back pairs cluster together and all technical replicate hybridizations cluster together when using the intrinsic genes. Sample identifiers have been colored according to the patient diagnosis: dSSc is red, lSSc is orange, morphea and EF are black, and normal controls are green (**Figure 2A**). The dendrogram has been colored to reflect the signatures of gene expression that are an inherent feature of the biopsies (**Figure 2A–C**).

The gene expression signatures further subdivide samples within existing clinical groups. We find a consistent set of genes that are highly expressed in a subset of the dSSc samples, which occupy the left branch of the dendrogram tree **(** **Figure 2D**). These groups have been labeled diffuse 1 (**Figure 2A**; blue branches) and diffuse 2 (**Figure 2A**; red branches) as they consistently cluster as two separate groups (c.f. **Figures 1** **and** **2**) and have distinct signatures of gene expression. The most consistent biological program expressed across the diffuse 1 and diffuse 2 scleroderma samples is that of proliferation (**Figure 2D**). We refer to this group broadly as *Diffuse-Proliferation*. A second group contains dSSc, lSSc and morphea samples on a single branch of the dendrogram tree (**Figure 2A**, purple branches). The genes most highly expressed in this group are those typically associated with the presence of inflammatory lymphocyte infiltrates as described above and this group has thus been labeled the *Inflammatory* group (**Figure 2F**). A third group contains primarily lSSc samples (*Limited*, orange branches, **Figure 2A**), which has low expression of the proliferation and T cell signatures but has high expression of a distinct signature found heterogeneously across the samples (**Figure 2C**). A branch of samples comprised primarily of healthy controls (green branches, **Figure 2A**) also contains samples from one patient with a diagnosis of dSSc and a patient with lSSc. This group has been labeled *Normal-like*, since the gene expression signatures in these samples more closely resemble and cluster with normal skin.

### Significance and reproducibility of intrinsic clustering

To examine the robustness of these groups, we performed two separate analyses: Statistical Significance of Clustering (SigClust)[57] and consensus clustering [58]. SigClust analysis was performed with the 995 intrinsic genes. At a p-value<0.001 we find five statistically significant clusters. The four major groups of *diffuse-proliferation*, *inflammatory*, *limited* and *normal-like* groups are each found to be statistically significant (**Figure 2A**); samples of patient dSSc8 form a statistically significant group of their own in the SigClust analysis (**Table 3**). Thus, the major groups identified in the hierarchical clustering using the 995 intrinsic genes are statistically significant and cannot be reasonably divided into smaller clusters with the current set of data. The two branches within the *diffuse-proliferation* group do not reach statistical significance in this analysis even though there are identifiable differences in their gene expression profile.

**Figure 3:**
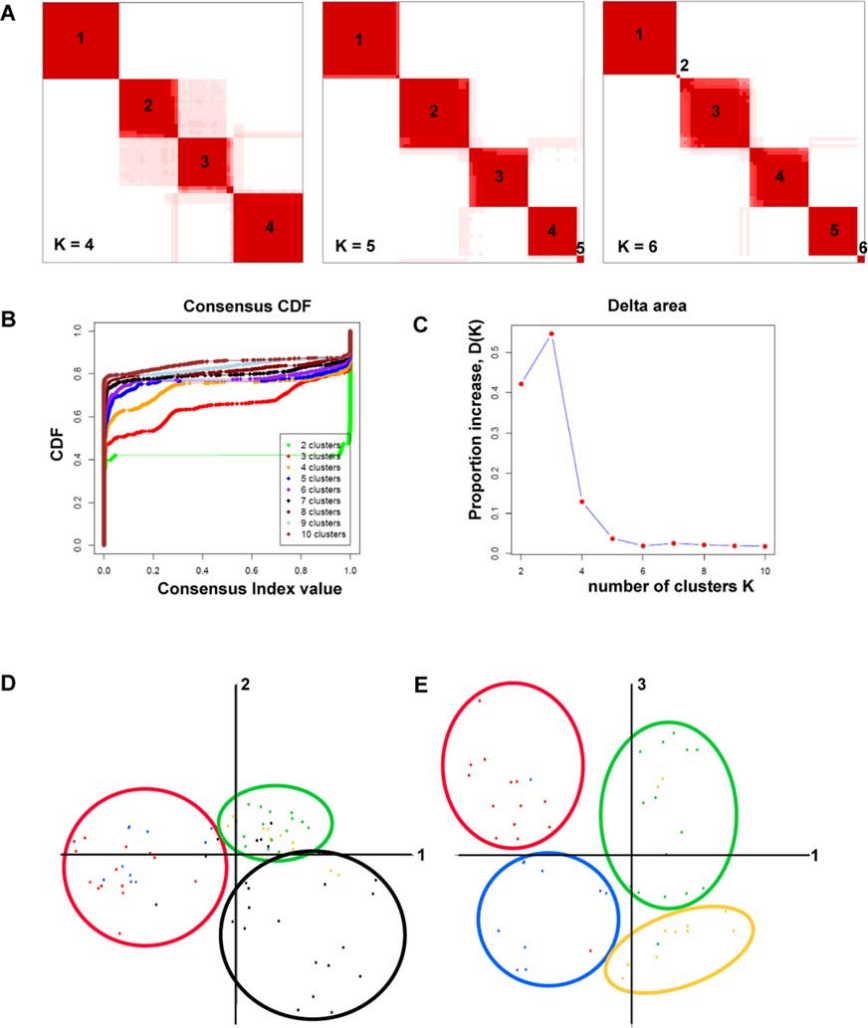
Robustness of sample classification. The robustness of the sample classifications was analyzed by consensus clustering, which uses multiple iterations of K-means clustering with random restart. 500 subsets of the data were sampled without replacement. The results of consensus clustering and Principal Component Analysis (PCA) applied to the 75 arrays and 995 intrinsic genes are shown. A. Consensus matrices are shown for K = 4, 5 and 6. Cluster numbers are shown and cluster assignments are summarized in Table 3. B. *Empirical consensus distribution function* (CDF) plots corresponding to K = 2,3,4…10. The ideal number of clusters can be identified when the area under the curve shows minimal increases with increasing K. C. Proportion increase *Δ(K)* in the area under the CDF. D. PCA was performed using TIGR MeV software; principal components 1 and 2 are plotted in 2-dimensional space. Samples (points in space) have been colored according Figure 2. *Normal-like* are green, *limited* orange, *diffuse-proliferation* in red and *inflammatory* in black. Circles indicate groups of samples distinguished by the top two principal components. E. Principal components 1 and 3 were plotted in two-dimensional space and show distinction between two groups within the *diffuse-proliferation*, *normal-like* and *limited* scleroderma.

To perform a second validation of the intrinsic groups, we used consensus clustering [58], which performs a K-means clustering analysis on randomly selected subsets of the data by resampling without replacement over 1,000 iterations using different values of *K*. **Figure 3A** shows the consensus over 1,000 iterations for K = 4, 5 and 6. To determine the number of clusters present in the data, we examined the area under the *Consensus Distribution Function* (CDF; **Figure 3B**). The point at which the area under the CDF ceases to show significant changes indicates the probable number of clusters (**Figure 3C**). The largest change occurs between three and four clusters with a slight change between four and five clusters (**Figure 3C**).

Based on this analysis and the SigClust analysis, we propose that there are approximately four to five statistically significant clusters in the data. The statistically significant cluster assignments from both SigClust and consensus clustering are summarized in **Table 3**. These are (1) *Diffuse-proliferation* comprised completely of patients with dcSSc, (2) *Inflammatory*, which includes a subset of dSSc, lSSc and morphea, (3) *Limited,* characterized by the inclusion of lSSc patients and (4) *Normal-like*, which includes five of six healthy controls along with two dSSc and one lSSc patients. Notably, three samples are not consistently classified into the primary clusters. These are: dSSc2 which is assigned to the either the *diffuse-proliferation*, *normal-like* or into a single cluster by itself, dSSc13 which is assigned to either *diffuse-proliferation* or the *limited* groups, and the patient EF which clusters either on the peripheral edge of the *diffuse-proliferation* cluster or is assigned to a cluster by itself.

To determine how sensitive the clustering was to the selection of the intrinsic genes, we analyzed the clustering results using a larger list of 2071 intrinsic genes and compared that clustering to that obtained with 995 intrinsic genes (**Supplemental Figure S3**). Although we find slight differences in the ordering of the samples, the major subsets of *diffuse-proliferation*, *inflammatory*, and *limited* are again identified. The *Normal-like* group is split onto two different branches using this larger set of genes. Samples that show inconsistent clustering are from patient dSSc2, dSSc8, dSSc13, and the single array for patient EF. The samples for each of these patients were also inconsistently classified in the SigClust and consensus clustering analysis using the 995 intrinsic gene set.

Principal Component Analysis (PCA) was used to confirm the sample grouping found by hierarchical clustering. PCA is an analytic technique used to reduce high dimensional data into more easily interpretable principal components by determining the direction of maximum variation in the data [59]. The 995 intrinsic genes were analyzed by PCA using the MultiExperiment Viewer (MeV) software [60]. The first and second principal components that capture the most variability in the data **(** **Figure 3D** **),** and the first and third principle components (**Figure 3E**) have been plotted in 2-dimensional space. The 2D projection shows that the samples group in a manner similar to that found by hierarchical clustering analysis: normal controls and limited samples group together and the two different groups of diffuse scleroderma group together (**Figure 3D**). Notably, the first and second principal components (**Figure 3D**) separate the *diffuse-proliferation*, the *inflammatory* and the *normal-like/Limited* groups. When the first and third principal components are analyzed (**Figure 3E**) we find that the distinction between *dSSc group 1* and *dSSc group 2* is clearly delineated, as is the distinction between *normal-like* and *limited*. The PCA analysis provides further evidence, in addition to our hierarchical clustering analysis, that the gene expression groups are stable features of the data.

### Biological processes differentially expressed in the intrinsic groups

**Figure 4:**
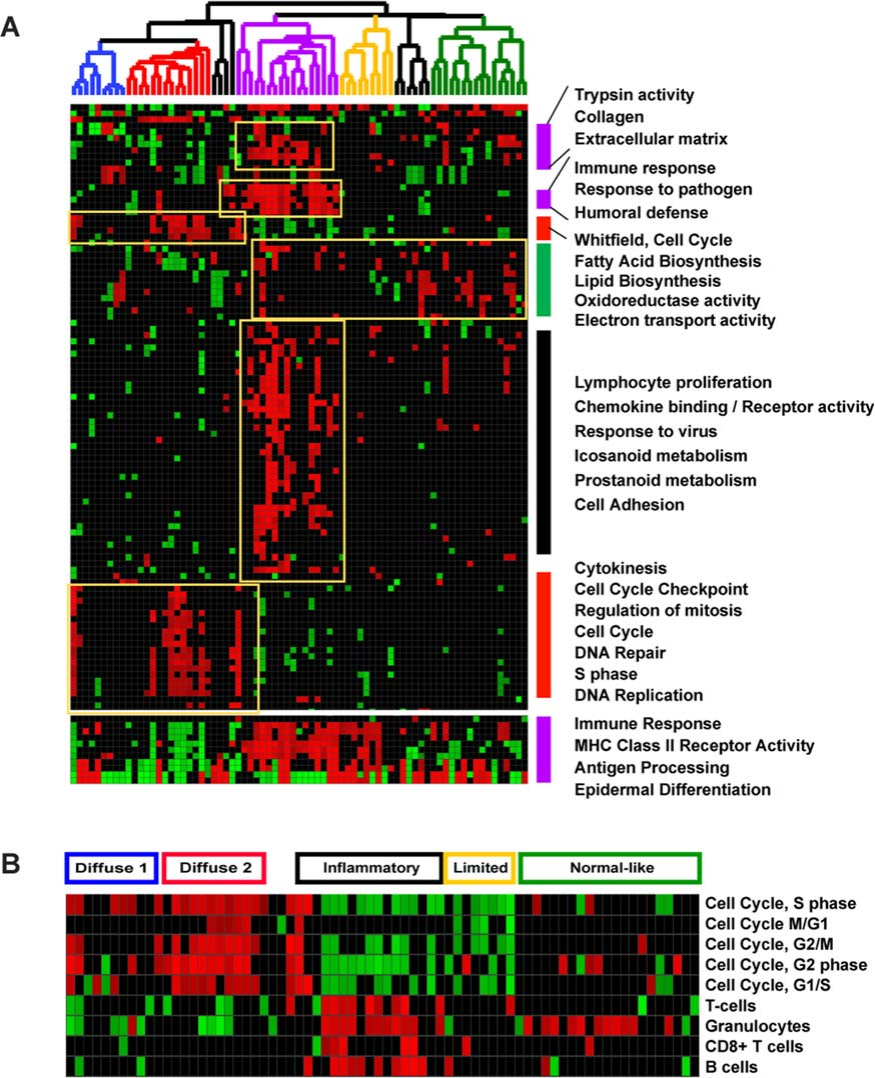
Scleroderma Module Map. A. Module map of the Gene Ontology (GO) Biological Processes differentially expressed among the scleroderma samples is shown. Each column represents a single microarray and each row represents a single GO Biological process. Patient samples are organized as described in Figure 2. Only modules that were significantly enriched (minimum 2-fold change, p<0.05) on at least 4 micoarrays are shown. The average expression of the gene hits from each enriched gene set is displayed here. Only gene sets that show significant differences after multiple hypothesis testing were included. Select GO biological processes are shown. The entire figure with all biological processes can be viewed in Supplementary Figure S4. B. Module map using gene list created from an experimental identification of all cell cycle-regulated genes [49] and genes found to be expressed in specific lymphocyte subsets [64].

In order to systematically investigate the biological processes found in the gene expression profiles of SSc, we created a module map using Genomica software [61], [62] (**Figure 4A**). A module map shows arrays that have co-expressed genes that map to specific gene sets. In this case, each gene set represents a specific biological process derived from Gene Ontology (GO) Biological process annotations [63], or from previously published microarray datasets [49], [64].

Modules with significantly enriched genes (p<0.05, hypergeometric distribution) and corrected for multiple hypothesis testing with an FDR of 0.1% are shown (**Figure 4A**). Expressed among the group *diffuse-proliferation* are the biological processes of *cytokinesis*, *cell cycle checkpoint*, *regulation of mitosis*, *cell cycle*, *DNA repair*, *S phase*, and *DNA replication,* consistent with the presence of dividing cells. Decreased in this group are genes associated with *fatty acid biosynthesis*, *lipid biosynthesis*, *oxidoreductase activity* and decreased *electron transport activity*. The decrease in genes associated with fatty acid and lipid biosynthesis is notable given the loss of subcutaneous fat observed in dSSc patients [4].

Expressed in the *inflammatory* group are biological processes indicative of an increased immune response, including the GO biological processes of *immune response*, *response to pathogen*, *humoral defense*, *lymphocyte proliferation*, *chemokine binding*, *chemokine receptor activity*, and *response to virus* (**Figure 4A**). Biological processes of *icosanoid* and *prostanoid metabolism,* which represents synthesis of prostaglandin lipid second messengers, have been associated with immune responses [65], found to be highly expressed in rheumatoid arthritis [66], [67], [68] and associated with severity in collagen-induced arthritis in mice [69], [70]. Also expressed in this group are processes associated with fibrosis including *trypsin activity*, *collagen* and *extracellular matrix*. The full figure with all differentially expressed biological processes is available as **Supplemental Figure S4**.

In order to better define the proliferation signature observed, we created gene sets representing the genes periodically expressed in the human cell division cycle as defined by Whitfield et al. [49] (**Figure 4B**). Gene sets were created that included the genes with peak expression at each of the five different cell cycle phases, G1/S, S, G2, G2/M and M/G1 [49]. The enrichment of each of these five gene sets was statistically significant (p<0.05 using the hypergeometric distribution) and more highly expressed in the *diffuse-proliferation* group (**Figure 4B**).

To better characterize the lymphocyte infiltrates we generated gene sets representing lymphocyte subsets using results reported by Palmer and coworkers [64]. Using isolated populations of lymphocytes and DNA microarray hybridization, the genes specifically expressed in different lymphocyte subsets were identified. Subsets included T cells (total lymphocyte and CD8+), B cells, and granulocytes. We found four of these gene sets, B cells, T cells, CD8+ T cells and granulocytes, to have a statistically significant over-representation in the *inflammatory* group (**Figure 4B**). This suggests the gene expression signature expressed in this group is determined by the presence of infiltrating lymphocytes and specifically implies the infiltrating cells include T cells, B cells and granulocytes. Although we do not have a gene expression signature representative of macrophages or dendritic cells in this analysis, the macrophage marker CD163 is highly expressed in this group, suggesting innate immune responses may play an important role in disease pathogenesis.

### Immunohistochemistry (IHC)

In order to verify that the gene expression reflected increased numbers of infiltrating lymphocytes or proliferating cells, we performed IHC for T cells (anti-CD3), B cells (anti-CD20) and cycling cells (anti-KI67). Summarized in **table 4** is a full enumeration of marker positive cells counted from representative fields of all biopsies analyzed by IHC, with the observer blinded to disease state. All IHC images for all three markers are available as **supplemental figure S5**. Analysis of biopsies from each of the major intrinsic groups confirmed the results found in the gene expression signatures. The presence of infiltrating T cells was confirmed in the *inflammatory* group (**Table 4**). The largest numbers of T cells were found in perivascular and perifollicular distributions, as well as in the dermis, of two dSSc patients (dSSc5, dSSc6) assigned to the *inflammatory* group (**Table 4**). IHC was also performed on skin biopsies from two patients with morphea (Morph1, Morph 3) and each showed large numbers of infiltrating T cells. Only a small number of T cells were observed in two healthy controls analyzed (Nor2 and Nor3). A slight increase in T cells was observed in a perivascular distribution in the four patients assigned to *diffuse-proliferation* (dSSc1, dSSc2, dSSc11, dSSc12; **Table 4**), which had a lower expression of the T cell signature.

**Table 4:** Immunohistochemical staining for KI67 and CD3 in the intrinsic subsets.

Patient	Assignment ^a^	KI67 Append	KI67 Epiderm	KI67 Derm	CD3 Append	CD3 Epiderm	CD3 Derm
Nor2	Normal-like	10	11	0	14	0	3
Nor3	Normal-like	0	11	0	22	0	0
	***Normal-like ^b^***	***5***	***11***	***0***	***18***	***0***	***1.5***
Morph3	Inflammatory	1	13	0	205	18	107
Morph1	Inflammatory	0	21	0	36	5	14
dSSc5	Inflammatory	4	11	0	68	1	5
dSSc6	Inflammatory	7	0	0	83	2	15
	***Inflammatory***	***3***	***11.3***	***0***	***98***	***6.5***	***35.3***
dSSc1	Prolif (2)	4	20	0	56	0	0
dSSc11	Prolif (2)	8	14	0	12	0	7
dSSc2	Prolif (1)	0	22	1	31	0	2
dSSc12	Prolif (1)	2	85	0	55	10	16
	***Prolif***	***3.5***	***35.3***	***0.3***	***38.5***	***2.5***	***6.3***

Shown is the summary of total counts per skin biopsy as determined by IHC staining for KI67, which stains cycling cells, and CD3, which stains T cells. Each biopsy was also analyzed for CD20 and only a small number of cells were found around dermal appendages for Morph3 (3), dSSc6 (2) and dSSc12 (2). All other samples were negative for CD20 cells. (Append = dermal appendages (hair follicles, vascular structures, eccrine glands); Epiderm = epidermis; Derm = dermis). a. Intrinsic group to which each sample was assigned. b. Average of total counts per category.

Few CD20+ B cells were observed in the SSc skin biopsies. The immunoglobulin gene expression signature was observed in eight diffuse patients (dSSc1, dSSc3, dSSc6, dSSc7, dSSc8, dSSc10, dSSc11, dSSc12) and one limited patient (1SSc7; **Figure 2F**). Of the six patients analyzed by IHC (dSSc1, dSSc2, dSSc5, dSSc6, dSSc11, dSSc12), two samples (dSSc1and dSSc12) showed small numbers of CD20+ B cells.

The presence of the proliferation signature is correlated with an increase in the mitotic index or number of dividing cells in microarray studies of cancer [46], [47], [48], [49], [71]. To confirm the presence of proliferating cells in the dSSc skin biopsies, we performed IHC staining for KI67, a standard marker of cycling cells. Analysis of skin from healthy controls (Nor2, Nor3), morphea (Morph1, Morph3), and diffuse patients in the *inflammatory* group (dSSc5, dSSc6), showed no proliferating cells in the dermis, and a small numbers of proliferating cells surrounding dermal appendages and in the epidermal layer (**Table 4**). In contrast, analysis of the skin from four patients in the *diffuse-proliferation* subgroup (dSSc1, dSSc2, dSSc11 and dSSc12) showed higher numbers of proliferating cells primarily in the epidermis (**Table 4**). Therefore, we conclude that the proliferation signature is likely the result of an increased number of proliferating cells in the epidermal compartment of the SSc skin biopsies. The identity of these cells is very likely to be keratinocytes.

### Intrinsic gene expression maps to identifiable clinical covariates

**Figure 5:**
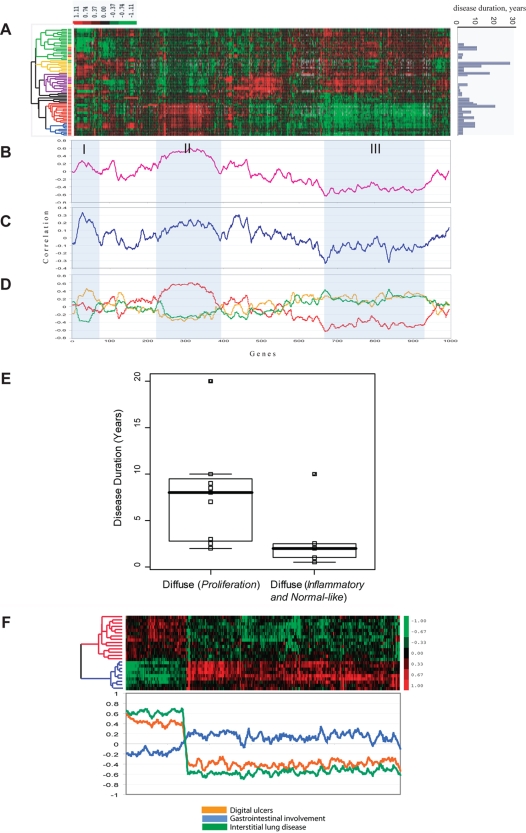
Correlation between gene expression and clinical covariates. A. Shown is the color-coded heatmap of the 75 arrays and 995 intrinsic genes. The graph on the right of the heat map shows disease duration for each sample. Disease duration was set to zero for normal controls and morphea samples. B. Pearson correlations were calculated between skin score and the expression values for each gene in the list. The moving average of the Pearson correlation (10-gene window) was plotted. Regions of high negative and high positive correlations to the three different clinical parameters are indicated (regions I–III shaded grey). C. Moving average of the Pearson correlation coefficients (10-gene window) between the self-reported Raynaud's severity score and the expression of each gene, D. Moving average of the Pearson Correlations (10-gene window) between extent of skin involvement and a diagnosis vector (see Methods) for dSSc(red), lSSc (orange) and healthy controls (green). E. Box plot of disease duration for dSSc patients. The patients included in the *diffuse-proliferation* group had disease duration of 8.4±6.4 years. The dSSc patients that fell into the *inflammatory* or *normal-like* groups have disease duration of 3.2±3.9 yrs (p<0.12, t-test). F. Genes that ideally discriminate ‘Diffuse 1’ and ‘Diffuse 2’ groups were selected using Significance Analysis of Microarrays (SAM). 329 genes were selected with an FDR<1%. Pearson correlation coefficients were calculated between each clinical parameter and the expression for each gene and plotted as a 10-gene moving window.

To map the intrinsic groups to specific clinical covariates, Pearson correlations were calculated between the gene expression of each of the 995 intrinsic genes and different clinical covariates. Shown are the results for three different covariates: the modified Rodnan skin score (MRSS; 0–51 scale), a self-reported Raynaud's severity score (0–10 scale), and the extent of skin involvement (dSSc, lSSc and unaffected). Each group was analyzed for correlation to each of the clinical parameters listed in Table 1; only the significant associations are shown. **Figure 5A** shows the gene expression patterns of the 995 intrinsic genes with each row representing a microarray and each column representing a gene. Pearson correlation coefficients were calculated between each of the clinical parameters and the expression of each gene. The moving average (10-gene window) of the resultant correlation coefficients is plotted for MRSS (**Figure 5B**), Raynaud's severity (**Figure 5C**) and degree of skin involvement (**Figure 5D**). Areas of high positive correlation between a clinical parameter and the expression of a group of genes indicate that increased expression of those genes is associated with an increase in that clinical covariate; a negative correlation indicates a relationship between a decrease in expression of the genes and an increase in a clinical covariate.

Areas of high positive or high negative correlation are highlighted in three different panels (**Figure 5B–D**, Regions I–III). Each of the three clinical covariates shows high positive correlations to a subset of gene expression signatures. Most notably, the MRSS skin score shows a high positive correlation to the ‘proliferation signature’ (**Figure 5B**, region II) with correlations ranging from 0.5 and 0.6. This signature is highly expressed in *diffuse-proliferation* samples but has low expression in the *inflammatory* group. The Raynaud's severity score has a high positive correlation to genes expressed at higher levels in the *limited* group and heterogeneously expressed in patients with dSSc (**Figure 5C**, region I). Not surprisingly, the genes highly correlated with MRSS also show a high positive correlation with diffuse skin involvement (**Figure 5D**, region II). While this signature associates with diffuse skin involvement, it is important to note that a subset of dSSc skin biopsies do not express this signature and have low skin scores. Similarly, the genes that have a high positive correlation with Raynaud's severity and a high positive correlation with the *limited* group (**Figure 5C**), which typically has more severe vascular involvement, are uncorrelated with the diagnosis of dSSc and are expressed at low levels in healthy control samples (**Figure 5D** **,** region III). Moving averages of the Pearson correlation between the intrinsic genes and other clinical covariates (digital ulcers, ILD, or GI involvement) were also calculated but did not reveal significant regions of positive or negative correlation to the gene expression profiles (data not shown).

One initial hypothesis was that there would be an obvious trend in the gene expression data reflecting the progressive nature of SSc in some patients. To examine this more carefully, disease duration in years since first onset of non-Raynaud's symptoms is plotted along the X-axis of the heat map (**Figure 5A**, right panel). The mean disease duration for the *diffuse-proliferation* group is 8.4±6.4 yrs, whereas mean disease duration for the *inflammatory* group, which includes dSSc and lSSc, is 6.5±6.1 yrs. Using a Student's t-test with a two-tailed distribution we find that this difference is not statistically significant. To test the hypothesis that a subset of the patients was grouping by disease duration, we analyzed the disease duration between the dSSc patients in the *diffuse-proliferation* group and the dSSc patients that were classified as either *inflammatory* or *normal-like* (**Table 3**). The *diffuse-proliferation* group has a mean disease duration of 8.4±6.4 years, and the dSSc patients in the *inflammatory* and *normal-like* groups have a mean disease duration of 3.2±3.9 yrs (**Figure 5E**, p = 0.12, t-test). The difference in the means between these two groups is clear, but outliers in each reduce the significance of the result. Dropping the two outliers results in p = 0.0042 (unequal variance two-sample t-test, two-sided)). Therefore, we conclude that there is a significant association between disease duration and the intrinsic groups for dSSc samples.

Since no obvious clinical covariate was identified that differentiated the dSSc group 1 from dSSc group 2, we selected the genes that most differentiated the two groups. Genes were selected that differentiated group 1 from group 2 using a non-parametric t-test implemented in Significance Analysis of Microarrays (SAM) [72]. 329 genes were selected that were differentially expressed between these two groups with an FDR of 0.19% (**Figure 5F** **; Supplementary Data File S3**). We analyzed these 329 genes for correlation to clinical covariates. Three clinical covariates were found associated with these two groups. The genes highly expressed in the dSSc group 2 (9 patients) are highly correlated with the presence of digital ulcers (DU) and the presence of interstitial lung disease (ILD) at the time the skin biopsies were taken. In contrast, dSSc group 1 (2 patients, both male) did not have DU or ILD at the time of biopsy. Although this grouping could result simply from stratification by sex, it also may reflect a true difference in disease presentation. Only 18 of the 329 genes map to either the X or Y chromosomes and thus are expected to be differentially expressed, suggesting the remainder may represent biology underlying these groups.

### A subset of genes is associated with increased modified Rodnan skin score

**Figure 6:**
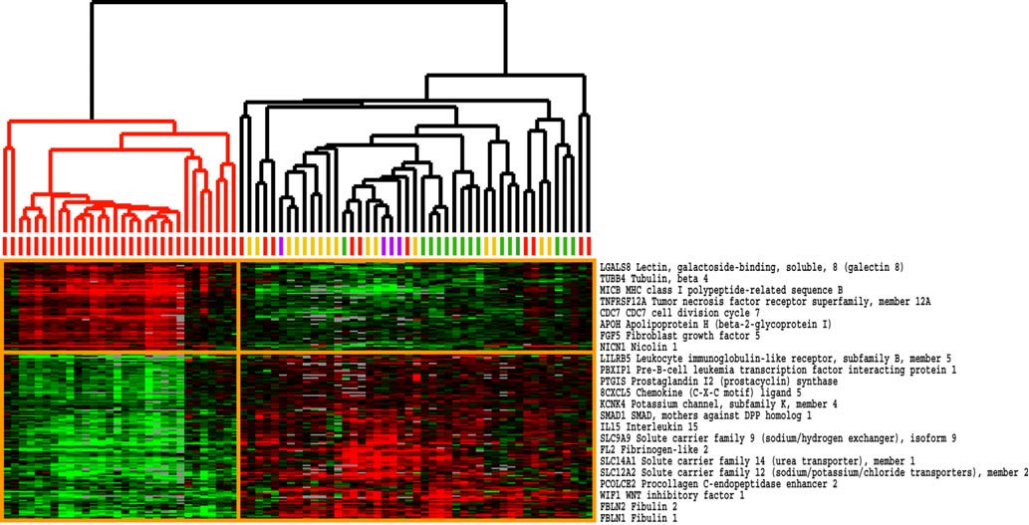
Genes correlated with MRSS. We selected the genes from the 995 intrinsic list that had a correlation greater than 0.5 or less than −0.5 to the MRSS. This list of 177 genes was then used to organize the skin biopsies. Forearm-back pairs from 14 patients with dSSc (mean MRSS of 26.34±9.42) clustered onto one branch of the dendrogram tree. The forearm-back pairs of 4 patients with dSSc (Mean MRSS 18.11±6.45) clustered onto a different branch of the dendrogram tree. The difference in skin score between these two groups is statistically significant (p<0.0197).

To identify genes associated with MRSS we selected the subset of genes most highly correlated with each covariate from the intrinsic list using Pearson correlations. 177 genes were selected from the 995 intrinsic genes that had Pearson correlations with MRSS >0.5 or <−0.5. We then used this list of 177 genes to organize the skin biopsies by average linkage hierarchical clustering (**Figure 6** **; Supplementary Data File S4**). We find that both forearm and back skin biopsies from 14 patients with dSSc (mean MRSS of 26.34±9.42) clustered onto a single branch of the dendrogram. All other samples, including the forearm-back pairs of 4 patients with dSSc (mean MRSS 18.11±6.45) clustered onto a separate branch of the dendrogram. Using a two-tailed Student's t-test we find that the difference in skin score between the two groups of dSSc is statistically significant (p = 0.0197).

From this analysis, 62 genes were expressed high levels and 115 genes were expressed at low levels in the patients with the highest skin score. Genes highly expressed include the cell cycle genes CENPE, CDC7 and CDT1, the mitogen Fibroblasts Growth Factor 5 (FGF5), the immediate early gene Tumor Necrosis Factor Receptor Superfamily member 12A (TNFRSF12A) and TRAF interacting protein (TRIP). Since skin score is considered to be an effective measure for disease outcome, this 177-gene group may contain genes that could be further developed into surrogate markers for skin score.

### Quantitative Real Time PCR

**Figure 7:**
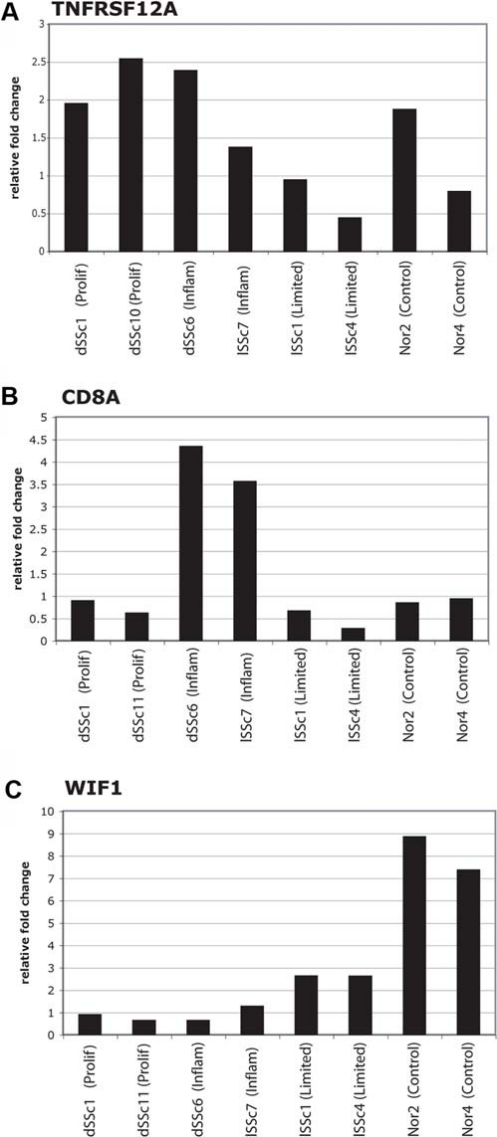
Quantitative Real Time PCR analysis of representative biopsies. The mRNA levels of three genes, TNFRSF12A (A), CD8A (B) and WIF1 (C) were analyzed by Taqman quantitative real time PCR. Each was analyzed in two representative forearm skin biopsies from each of the major subsets of proliferation, inflammatory, limited and normal controls. In the case of TNFRSF12A, patient dSSc11 was replaced by patient dSSc10, which cluster next to one another in the intrinsic subsets and show similar clinical characteristics (Table 1). Each qRT-PCR assay was performed in triplicate for each sample. The level of each gene was then normalized against triplicate measurements of GAPDH to control for total mRNA levels (see materials and methods). The relative expression values are displayed as the fold change for each gene relative to the median value of the eight samples analyzed.

In order to validate the gene expression in the major groups found in this study, we performed quantitative real time PCR (qRT-PCR) on three genes selected from the intrinsic subsets (**Figure 7**). These included TNFRSF12A, which is highly expressed in the dSSc patients and shows high expression in patients with increased MRSS (see **Figure 6**), WIF1, which shows low expression in SSc its decreased expression is associated with increased MRSS, and CD8A, which is highly expressed in CD8+ T cells and is highly expressed in the inflammatory subset of patients. A representative sampling of patients from the intrinsic subsets was analyzed for expression of these three genes. Each was analyzed in triplicate and standardized to the expression of GAPDH.

Each gene is shown with the fold change relative to the median value for the eight samples analyzed. TNFRSF12A shows highest expression in the patients with dSSc and the lowest in patients with limited SSc and normal controls. The three patients with highest expression are dSSc and include the proliferation group (**Figure 7A**). CD8A shows highest expression in the inflammatory subgroup as predicted by our gene expression subsets (**Figure 7B**). WIF1 shows highest expression in the healthy controls with approximately 4–8 fold relative decrease in patients with SSc (**Figure 7C**). The most dramatic decrease is in patients with dSSc with smaller fold changes in patients with lSSc.

## Discussion

We have used DNA microarrays to determine if the heterogeneity in scleroderma can be captured quantitatively and objectively using gene expression profiling. We used an experimental design that has previously been used with great success to identify molecular subsets in tumors [47], [48], [56], [73], [74], [75] and now shows that we can also find subsets in the gene expression patterns of scleroderma, a disease of completely different etiology but also characterized by disease heterogeneity.

Our results show that the diversity in the gene expression patterns of SSc is much greater than demonstrated in two prior studies of dSSc skin [16], [17]. We find evidence for four major groups, each characterized by a distinct gene expression profile. The *diffuse-proliferation* group is composed solely of patients with a diagnosis of dSSc, the *inflammatory* group includes patients with dSSc, lSSc and morphea, the *limited* group is comprised solely of patients with lSSc, and the *normal-like* group includes healthy controls along with dSSc and lSSc patients. The *diffuse-proliferation* group contains two potential subgroups, however, our sample size is not large enough to draw definitive conclusions regarding their stability.

It is unlikely that the underlying gene expression groups result from technical artifacts or heterogeneity at the site of biopsy. First, we created a standardized sample-processing pipeline, which was extensively tested on skin collected from surgical discards prior to beginning this study and included strict protocols that were used throughout with the goal of eliminating variability in sample handling and preparation. Second, all gene expression groups were analyzed for correlation to date of hybridization, date of sample collection and other technical variables that might have affected the groupings. Also, heterogeneity at the site of biopsy is unlikely to account for the findings as the signatures used to classify the samples were selected by virtue of their being expressed in both the forearm and back samples of each patient. The inflammatory group is unlikely to be a result of active infection in patients as individuals with active infections were excluded from the study. Finally, the gene expression signatures we found are supported by both the IHC findings (**Table 4**) and the quantitative real-time PCR findings (**Figure 7**).

We were able to associate our gene expression signatures with changes in specific cell markers. We have confirmed infiltration of T cells in the dermis of the ‘inflammatory’ subgroup, and have confirmed an increase in the number of proliferating cells in the epidermis in the ‘proliferation’ group. The increase in the number of proliferating cells in the epidermis could result from paracrine influences on the resident keratinocytes, possibly activated by the profibrotic cytokine TGFβ. We were not able to find significant numbers of CD20 positive B-cells.

An open question that remains is how do these gene expression changes correlate with more specific histological changes in the skin? Two studies of gene expression in liver [76] and in the brain [77] have correlated large-scale morphological changes with the changes in gene expression. In each case it was possible to create a detailed map linking gene expression to features in detailed imaging analysis providing addition insight into tumorigenesis. A comprehensive gene expression study in SSc that combines detailed histological or morphological analysis of fat changes, vascular changes and dermal markers, would provide additional insight into how the gene expression changes correlate with morphological changes in SSc skin. Unfortunately these analyses are not possible with our current set of data.

The detection of subsets in the gene expression of SSc raises questions as to their etiology. Do these subsets represent distinct groups with stable patterns of gene expression or do the groups represent different time-dependent phases of the disease? We have found a clear relationship between severity of disease and gene expression (**Figures 6** **–** **7**), but only a weak association between duration of disease and gene expression (**Figure 6B**). However, analysis of disease duration in only the dSSc patients raises the possibility that the groups we have labeled as *inflammatory* and *normal-like* include patients in the early stages of disease, while the diffuse-proliferation group includes patients with later stage disease. There is the distinct possibility that patients with the *inflammatory* gene expression signature will eventually progress to a gene expression signature more characteristic of the *diffuse-proliferation* group - a hypothesis that can only be addressed directly in a longitudinal study of a well-defined patient cohort.

The multiple groups observed in our gene expression data may correspond to patients that will have distinct clinical outcomes. This is supported by recent work analyzing the relationship between change in skin score and outcome in a large single center cohort of 225 patients [14]. Using a Latent linear trajectory model, Denton and coworkers were able to classify 58% of their patients into 1 of 3 subgroups with different skin score trajectories. Each group showed different progression to clinical endpoints. Survival was lowest in a group with the highest baseline skin score and showed little improvement during follow-up. A second group had severe MRSS but improved with follow-up and a third group had low initial MRSS and subsequent improvement. A second study analyzed SSc patients with anti-topoisomerase I (anti-topo I) antibodies and found patients could be divided into five different subgroups based on skin thickness progression rates [6]. These included three groups of dSSc patients and two groups of lSSc patients.

This study allows us to then propose two different models that could account for the gene expression subsets we have found in scleroderma. The first model is that there are multiple distinct groups of scleroderma patients, each exhibiting distinct gene expression profiles. The aberrant gene expression patterns may be established early in the disease and remain stable during disease progression. In this case, serial biopsies taken over time would result in sequential biopsies from the same patient always remaining in the same group. It would likely be possible to identify the clinical endpoints and complications to which each group would progress. The implications are that it may be possible to predict patient outcome based on their gene expression profile. The reports of three different groups of diffuse patients with different outcome trajectories or different skin thickness progress rates supports this model [6], [14].

The second model is that the different gene expression subgroups represent different disease stages. This is supported in part by the analysis of disease duration since the first onset of non-Raynaud's symptoms between the group we labeled *diffuse-proliferation*, and the dSSc patients that were classified as either *inflammatory* or *normal-like* (**Figure 5E**). There is an obvious trend toward the patients in the very earliest stages of disease mapping to the *inflammatory* group and the latest stage patients mapping to the *diffuse-proliferation* group.

The gene expression profiles in scleroderma hold the promise of identifying markers of disease activity that could be used as surrogate markers in clinical trials. Therefore, the analysis of skin biopsies before and after treatment may be useful in testing the efficacy of novel therapeutics. To this end, we have identified 177 genes that are strongly correlated with the severity of skin disease. These genes may point to a novel pathway involved in skin fibrosis that includes TNFRSF12A (Tweak Receptor (TweakR); Fn14), which is a TNF receptor family member expressed on both fibroblasts [78] and in endothelial cells [79]. It is induced by FGF1 and other mitogens, including the proinflamatory cytokine TGFβ (J.L.S. and M.L.W., unpublished). In fibroblasts, increased expression results in decreased adhesion to ECM proteins fibronectin and vitronectin [78]. TNFRSF12A has also been shown to play role in angiogenesis [79]. In vitro cross-linking of the TNFRSF12A in endothelial cells stimulates endothelial cell proliferation [79], while inhibition prevented endothelial cell migration in vitro and angiogenesis in vivo. Activation of TNFRSF12A in human dermal fibroblasts results in increased production of MMP1, the proinflammatory prostaglandin E2, IL6, IL8, RANTES and IL10 [80]. The cytoplasmic domain of TNFRSF12A binds to TRAF1, 2 and 3 [79]. A factor downstream of the TRAFs, TRIP (TRAF Interacting Protein), is highly correlated with MRSS. With further refinement, these genes could serve as surrogate markers for disease severity in scleroderma.

## Materials and Methods

Ethics approval was obtained for this study from the University of California at San Francisco's Committee on Human Research (CHR) and from Dartmouth College's Committee for the Protection of Human Subjects (CPHS). All subjects signed consent forms approved by the CHR at the University of California, San Francisco (UCSF). All patients met the American College of Rheumatology classification criteria for SSc [8] and were further characterized as the diffuse (dSSc) [3], or the limited (lSSc) subsets [1]. LSSc patients had 3 of the 5 features of CREST (calcinosis, Raynaud's syndrome, esophageal dysmotility, sclerodactyly and telangiectasias) syndrome, or had Raynaud's phenomenon with abnormal nail fold capillaries and scleroderma-specific autoantibodies. The diffuse systemic sclerosis (dSSc) had wide spread scleroderma and MRSS ranging from 15 to 35. The lSSc patients had MRSS ranging from 8 to 12. Patients with undifferentiated connective tissue disease (UCTD) were excluded from the study.

Skin biopsies were taken from a total of 34 individuals: 17 patients with dSSc, 7 patients with lSSc, 3 patients with morphea (MORPH), 6 healthy volunteers (NORM) and one patient with eosinophilic fasciitis (EF) (**Table 1**). dSSc patients (median age 49±9.4 years) were divided into two groups by their disease duration as defined by first onset of non-Raynaud's symptoms. Eight of the dSSc patients had disease duration <3 years since onset of non-Raynaud's symptoms (median disease duration 2.25±0.8 years) and nine dSSc patients had disease duration >3 years since onset of non-Raynaud's symptoms (median disease duration 9±5.3 years). The seven patients with lSSc had a median disease duration 5±9.7 years. The three patients with morphea had median disease duration 7±6.2 years.

In most cases, two 5-mm punch biopsies were taken from the lateral forearm, 8 cm proximal to the ulna styloid on the exterior surface non-dominant forearm for clinically involved skin. Two 5-mm punch biopsies were also taken from the lower back (flank or buttock) for clinically uninvolved skin. Thirteen dSSc patients provided forearm and back biopsies; four dSSc patients provided only single forearm biopsies. The seven lSSc patients and all six healthy controls also underwent two 5-mm punch biopsies at the identical forearm and back sites. Three subjects with morphea underwent two 5-mm punch biopsies at the clinically affected areas of the leg (MORPH1), abdomen (MORPH2), and back (MORPH3).

For each patient, one biopsy was immediately stored in 1.5. mL RNAlater (Ambion) and frozen at −80°C, a second biopsy was bisected; half went into 10% formalin for routine histology and half was fresh frozen. In total, 61 biopsies were collected for microarray hybridization: 30 from dSSc, 14 from lSSc, 4 from morphea, 1 eosinophilic fasciitis, and 12 from healthy controls (**Table 2**).

RNA was prepared from each biopsy by mechanical disruption with a PowerGen125 tissue homogenizer (Fisher Scientific) followed by isolation of total RNA using an RNeasy Kit for Fibrous Tissue (Qiagen). Approximately 2–5 µg of total RNA was obtained from each biopsy.

### cRNA synthesis, microarray hybridization and data processing

200 ng of total RNA from each biopsy was converted to Cy3-CTP (Perkin Elmer) labeled cRNA, and Universal Human Reference (UHR) RNA (Stratagene) was converted to Cy5-CTP (Perkin Elmer) labeled cRNA using a low input linear amplification kit (Agilent Technologies). Labeled cRNA targets were then purified using RNeasy columns (Qiagen). Cy3-labeled cRNA from each skin biopsy was competitively hybridized against Cy5-CTP labeled cRNA from Universal Human Reference (UHR) RNA pool, to 44,000 element DNA oligonucleotide microarrays (Agilent Technologies) representing more than 33,000 known and novel human genes in a common reference design [81]. Hybridizations were performed for 17 hours at 65°C with rotation.

After hybridization, arrays were washed following Agilent 60-mer oligo microarray processing protocols (6× SSC, 0.005% Triton X-102 for 10 min. at room temperature; 0.1× SSC, 0, 005% Triton X-102 for 5 min at 4°C, rinse in 0.1× SSC). Microarray hybridizations were performed for each RNA sample resulting in 61 hybridizations. Fourteen replicate hybridizations were added, resulting in a total of 75 microarray hybridizations.

Microarrays were scanned using a dual laser GenePix 4000B scanner (Axon Instruments). The pixel intensities of the acquired images were then quantified using GenePix Pro 5.0 software. Arrays were visually inspected for defects or technical artifacts, and poor quality spots were manually flagged and excluded from further analysis. Only spots with fluorescent signal at least twofold greater than local background in both Cy3- and Cy5- channels were included in the analysis. Probes missing more than 20% of their data points were excluded, resulting in 28,495 probes that passed the filtering criteria. The data were displayed as log_2_ of the LOWESS-normalized Cy5/Cy3 ratio. Since a common reference experimental design was used, each probe was centered on its median value across all arrays.

### Selection of intrinsic genes

An intrinsic gene identifier algorithm was used to select a set of intrinsic scleroderma genes. Detailed methods on the selection of intrinsic genes are available in [47] and http://genome-www.stanford.edu/breast_cancer/molecularportraits/. A gene was considered ‘intrinsic’ if it showed the most consistent expression between forearm-back pairs and technical replicates for the same patient, but had the highest variance in expression across all samples analyzed. The intrinsic gene identifier computes a weight for each gene, which is inversely related to how intrinsic the gene's expression is across the samples analyzed. A lower weight equals a higher ‘intrinsic’ character. A total of 34 experimental groups were defined, each representing the 34 different subjects in our study. Replicate hybridizations for a given patient were assigned to the same experimental group.

In order to estimate False Discovery Rate (FDR) at a given intrinsic weight, the analysis was repeated on data randomized in rows (i.e. across each gene). The FDR at a given weight was estimated by determining the number of genes that received the same weight or lower in the randomized data. 995 genes were selected that had an intrinsic weight <0.3; in randomized data 39±7 genes (calculated from 10 independent randomizations) had a weight of 0.3 or less, resulting in an FDR of approximately 4%. We found that a cutoff of 0.3 balanced the number of genes selected with an acceptable FDR, while retaining reproducible hierarchical clustering of technical replicate samples. Although it is possible to select a more or less restrictive list of genes with FDRs of 5% (weight <0.35; 2071 genes), 3.4% (weight <0.25; 425 genes) or 2.4% (weight <0.20; 171 genes), these smaller lists of genes resulted in less reproducible hierarchical clustering suggesting overfitting (**Supplemental Figure S3**).

### Hierarchical clustering

Average linkage hierarchical clustering was performed in both the gene and experiment dimensions using either Cluster 3.0 software (http://bonsai.ims.u-tokyo.ac.jp/mdehoon/software/cluster/software.htm) or X-Cluster (Gavin Sherlock; http://genetics.stanford.edu/sherlock/cluster.html) using Pearson correlation (uncentered) as a distance metric [18]. Clustered trees and gene expression heat maps were viewed using Java TreeView Software (http://jtreeview.sourceforge.net/) [82].

### Robustness and statistical significance of clustering

The statistical significance of clustering was assessed using Statistical Significance of Clustering (SigClust) [57] and Consensus Cluster [58]. SigClust tests the null hypothesis that the samples form a single cluster. A statistically significant p-value indicates the data came from a non-Gaussian distribution and that there is more than one cluster. Two different p-values were used to identify significant clusters, p<0.01 and p<0.001. The statistical significance of the clusters was first assessed at the root node of the tree derived from hierarchical clustering with the 995 intrinsic genes. If the cluster was statistically significant, the next node further down the tree was tested. The process ended when a cluster had a p-value greater than the established cutoff.

In addition, we analyzed the 995 intrinsic genes using Consensus Cluster [58]. Consensus Cluster is available through GenePattern (v.1.3.1.114; [83]). Assessment of sample clustering was performed by consensus clustering with *K* clusters (K = 2, 3, 4…10) using 1000 iterations with random restart. Samples that clustered together most often in each of the K clusters received a correlation value. The resulting consensus matrix was visualized as a color-coded heat map with varying shades of red, the brighter of which corresponded to higher correlation among samples (**Figure 3A**). Summary statistics are shown, including the *empirical consensus distribution function* (CDF) vs. the *consensus index value* (**Figure 3B**). Also shown are the proportion change *(ΔK)* under the CDF for each K = 2, 3…10 (**Figure 3C**). Consensus Cluster assignments for each sample are summarized in **Table 3**.

### Principal Component Analysis

Principal Component Analysis was performed using Multiexperiment Viewer (MeV) software version 4.0.01 (http://www.tm4.org/mev.html; [60]). Data was loaded into MeV as a tab delimited text file of log2-transformed Cy3/Cy5 ratios. For PCA analysis [59], missing data were first estimated using K-nearest neighbors (KNN) imputation with N = 4.

### Module Maps

Module maps were created using the Genomica software package [61], [62]. Gene sets containing all human Gene Ontology (GO) Terms were obtained from the Genomica web site (http://genomica.weizmann.ac.il/; Human_go_process.gxa, created Nov. 20, 2006). Additional custom gene sets representing the human cell division cycle [49] and lymphocyte subsets [64] were created specifically for this study. The human cell division cycle gene set was created from the genes found to periodically expressed in human HeLa cells [49]. Genes found to show peak expression at the five different cell cycle phases G1/S, S, G2, G2/M and M/G1 were each put into their own independent gene list. Gene sets representing different lymphocyte populations, T cells (total population, CD4+, CD8+), B cells, and granulocytes, were derived for this study from the genes expressed in isolated lymphocyte subsets by Palmer and coworkers [64].

All 75 microarray experiments and 28,495 DNA probes were included in the module map analysis. The 28,495 probes were collapsed to 14,448 unique LocusLink Ids (LLIDs) [84]. Only gene sets with at least three genes but fewer than 1000 genes were analyzed. A gene set was considered enriched on a given array if at least 3 genes from that set were considered to be significantly up-regulated or down-regulated (minimum 2-fold change, p<0.05, hypergeometric distribution) on at least four microarrays. Each gene set was corrected for multiple hypothesis testing using an FDR correction of 0.1%.

### Correlation to clinical parameters

Pearson correlations were calculated between each clinical parameter and the gene expression data in Microsoft Excel. Pearson correlations between the diagnosis of dSSc, lSSc and healthy controls and the gene expression data were calculated by creating a ‘diagnosis vector’. The diagnosis vector was created by assigning a value 1.0 to all dSSc samples and 0.0 to all remaining samples for the dSSc vector; lSSc and healthy controls were treated similarly creating a vector for each. Pearson correlations were calculated between the gene expression vector and the diagnosis vector for dSSc, lSSc and healthy controls. Correlations between the gene expression and clinical data were plotted as a moving average of a 10-gene window.

### Immunohistochemistry (IHC)

IHC was performed on paraffin embedded sections at the University of California, San Francisco in the Immunohistochemistry and Molecular Pathology core facility. All immunostaining was completed via a semi-automated protocol utilizing an automated immunostainer (DAKO Corp, Carpenteria, CA). Slides were heated, deparaffinized and then hydrated. Protease digestion was completed followed by antigen retrieval via pressure cooker as per standard protocols. After an endogenous peroxidase block with 3% H_2_0_2_, slides were loaded on to the automated immunostainer. A primary antibody cycle of 30 min was followed by a secondary antibody cycle using the ENVISION+ system. Color development was completed using DAB followed by counterstaining with Gills #2 Hematoxylin. Specific conditions for the antibodies utilized were as follows: anti-CD20 (DAKO) was used at 1∶600 for 30 minutes in citrate buffer (pH 6.0); anti-CD3 (DAKO) at 1∶400 for 30 minutes in Tris buffer (pH 9.0), and anti-Ki67 (MiB1; DAKO) was used at 1∶1000 for 30 minutes in Tris buffer (pH 9.0). Marker positive cells were enumerated by tissue compartment in equal sized images of *n* skin biopsies, with the observer blinded to disease state and array results of the specimens (**Table 4**).

#### Quantitative Real-Time PCR

Each quantitative real time PCR assay [85] was performed with 100–200 ng of total RNA. Each sample was reverse-transcribed into single-stranded cDNA using SuperScript II reverse transcriptase (Invitrogen, San Diego, CA). 96-well optical plates were loaded with 25 µl of reaction mixture which contained: 1.25 µl of TaqMan® pre-designed Primers and Probes, 12.5 µl of TaqMan® PCR Master Mix, and 1.25 ng of cDNA. Each measurement was carried out in triplicate with a 7300 Real-Time PCR System (Applied Biosystems). Each sample was analyzed under the following conditions: 50°C for 2 min and 95°C for 10 min, and then cycled at 95°C for 15 sec and 60°C for 1 min for 40 cycles. Output data was generated by the instrument onboard software 7300 System version 1.2.2 (Applied Biosystems). The number of cycles required to generate a detectable fluorescence above background (C_T_) was measured for each sample. Fold difference between the initial mRNA levels of target genes (TNFRSF12A, CD8A and WIF1) in our experimental samples were calculated with the comparative C_T_ method using formula 2^−ΔΔCT^ [86] and median centered across all samples analyzed.

### Data Access

The full dataset, figures in both red/green and blue/yellow format, as well as searchable versions of figures 1 and 2 are available at website maintained by the authors: http://whitfieldlab.dartmouth.edu/SScSubsets/. Raw data is available from the UNC Microarray Database (https://genome.unc.edu/) and has been deposited to NCBI's Gene Expression Omnibus (GEO; http://www.ncbi.nlm.nih.gov/geo/; accession Number: GSE9285).

## Supporting Information

Figure S1 Gene expression signatures in scleroderma. 4,149 probes that changed at least 2-fold from their median value on at least two microarrays were selected from 75 microarray hybridizations representing 61 biopsies. Probes and microarrays were ordered by 2-dimensional average linkage hierarchical clustering. This clustering shows that the dSSc, lSSc, morphea samples form distinct groups largely stratified by their clinical diagnosis. A. The unsupervised hierarchical clustering dendrogram shows the relationship among the samples using this list of 4,149 probes. Samples names have been color-coded by their clinical diagnosis: dSSc in red, lSSc in orange, morphea and EF in black, and healthy controls (Nor) in green. Forearm (FA) and Back (B) are indicated for each sample. Solid arrows indicate the 14 of 22 forearm-back pairs that cluster next to one another; dashed arrows indicate the additional 3 forearm-back pairs that cluster with only a single sample between them. Technical replicates are indicated by the labels (a), (b) or (c). 9 out of 14 technical replicates cluster immediately beside one another. B. Overview of the gene expression profiles for the 4,149 probes. Each probe has been centered on its median expression value across all samples analyzed. Measurements that are above the median are colored red and those below the median are colored green. The intensity of the color is directly proportional to the fold change. Groups of genes on the right hand side indicated with colored bars are shown in greater detail in panels C – H. C. Immunoglobulin genes expressed highly in a subset of patients with dSSc and in patients with morphea, D. proliferation signature, E. collagen and extracelluar matrix components, F. genes typically associated with the presence of T-lymphocyes and macrophages, G. Genes showing low expression in dSSc, H. Heterogeneous expression cluster that is high in lSSc and a subset of dSSc. This figure shows all gene names associated with the panels in figure 1 and is designed to be viewed in a digital format only so that one can zoom in to read the gene names.
(3.00 MB PDF)

Figure S2 Cluster analysis using the scleroderma intrinsic gene set. The 995 most ‘intrinsic’ genes selected from 75 microarray hybridizations analyzing 34 individuals. Two major branches of the dendrogram tree are evident which divide a subset of the dSSc samples from all other samples. Within these major groups are smaller branches with identifiable biological themes, which have been colored accordingly: blue for diffuse 1, red for diffuse 2, purple for inflammatory, orange for limited and green for normal-like. Statistically significant clusters (p<0.001) identified by SigClust are indicated by an asterisk (*) at the lowest significant branch. A. Experimental sample hierarchical clustering dendrogram. Black bars indicate forearm-back pairs which cluster together based on this analysis. B. Scaled down overview of the intrinsic gene expression signatures. C. Limited SSc gene expression -cluster. D. Proliferation cluster. E. Immunoglobulin gene expression cluster. F. T-lymphocyte and IFNγ gene expression cluster. This file shows all gene names associated with the panels in figure 2 and is designed to be viewed in a digital format only so that one can zoom in to read the gene names.
(6.69 MB PDF)

Figure S3 Robustness of intrinsic clustering. Hierarchical clustering was performed with two different sets of intrinsic genes. A. 995 intrinsic genes (weight <0.3; 4% FDR), B. 2071 intrinsic genes (weight <0.35, 5% FDR). Statistically significant clusters (p<0.05) as determined by SigClust are indicated by an asterisk (*). Transparent bars indicate the movement of groups of samples. The major clusters are recapitulated with this larger set of genes.
(1.07 MB TIF)

Figure S4 Scleroderma Module Map. Module map of the Gene Ontology (GO) Biological Processes differentially expressed among the scleroderma samples is shown. Each column represents a single microarray and each row represents a single GO Biological process. Patient samples are organized as described in Figure 2. Only modules that were significantly enriched (minimum 2-fold change, p<0.05) on at least 4 micoarrays are shown. The average expression of the gene hits from each enriched gene set is displayed here. Only gene sets that show significant differences after multiple hypothesis testing were included. This figure is best viewed in PDF format in order to read all modules names.
(2.41 MB TIF)

Figure S5 Immunohistochemistry for lymphocyte subsets and proliferating cells in scleroderma skin. Lymphocyte subsets in forearm biopsies of six dSSc patients, the leg and back specimens of two morphea patient and forearm samples of two healthy control were analyzed by immunohistochemistry. Paraffin sections were stained for T cells (CD3), B cells (CD20) and proliferating cells (KI67). (Magnification: ×200). See table 4 for detailed quantification.
(9.60 MB TIF)

Data File S1 4,149 probes shown in figure 1 that changed at least 2-fold from their median value on at least two microarrays were selected from 75 microarray hybridizations representing 61 biopsies.
(2.44 MB TXT)

Data File S2 995 intrinsic genes shown in figure 2. An intrinsic gene identifier algorithm was used to select a set of intrinsic scleroderma genes. A gene was considered ‘intrinsic’ if it showed the most consistent expression between forearm-back pairs and technical replicates for the same patient, but had the highest variance in expression across all samples analyzed.
(0.59 MB TXT)

Data File S3 Supporting data file for figure 5F. Genes the differentiate dSSc group 1 vs. group 2
(0.09 MB TDS)

Data File S4 Supporting data file for figure 6. Included are the 177 genes correlated with modified rodnan skin score.
(0.10 MB TXT)

## References

[1] Mayes MD (1998). Classification and epidemiology of scleroderma.. Semin Cutan Med Surg.

[2] Mayes MD, Lacey JV, Beebe-Dimmer J, Gillespie BW, Cooper B (2003). Prevalence, incidence, survival, and disease characteristics of systemic sclerosis in a large US population.. Arthritis Rheum.

[3] Leroy EC, Black C, Fleischmajer R, Jablonska S, Krieg T (1988). Scleroderma (systemic sclerosis): classification, subsets and pathogenesis.. JRheumatol.

[4] Medsger TA, Koopman WJ (2001). Systemic sclerosis (scleroderma): clinical aspects.. Arthritis and Allied Conditions. 14th ed.

[5] Masi AT (1988). Classification of systemic sclerosis (scleroderma): relationship of cutaneous subgroups in early disease to outcome and serologic reactivity.. J Rheumatol.

[6] Perera A, Fertig N, Lucas M, Rodriguez-Reyna TS, Hu P (2007). Clinical subsets, skin thickness progression rate, and serum antibody levels in systemic sclerosis patients with anti-topoisomerase I antibody.. Arthritis Rheum.

[7] Steen VD, Medsger TA (2000). Severe organ involvement in systemic sclerosis with diffuse scleroderma.. Arthritis Rheum.

[8] Committee. SfSCotARADaTC (1980). Preliminary criteria for the classification of systemic sclerosis (scleroderma)..

[9] Seibold JR, McCloskey DA (1997). Skin involvement as a relevant outcome measure in clinical trials of systemic sclerosis.. Curr Opin Rheumatol.

[10] Clements PJ, Hurwitz EL, Wong WK, Seibold JR, Mayes M (2000). Skin thickness score as a predictor and correlate of outcome in systemic sclerosis: high-dose versus low-dose penicillamine trial.. Arthritis Rheum.

[11] Clements PJ, Lachenbruch PA, Ng SC, Simmons M, Sterz M (1990). Skin score. A semiquantitative measure of cutaneous involvement that improves prediction of prognosis in systemic sclerosis.. Arthritis Rheum.

[12] Barnett AJ, Miller MH, Littlejohn GO (1988). A survival study of patients with scleroderma diagnosed over 30 years (1953–1983): the value of a simple cutaneous classification in the early stages of the disease.. J Rheumatol.

[13] Scussel-Lonzetti L, Joyal F, Raynauld JP, Roussin A, Rich E (2002). Predicting mortality in systemic sclerosis: analysis of a cohort of 309 French Canadian patients with emphasis on features at diagnosis as predictive factors for survival..

[14] Shand L, Lunt M, Nihtyanova S, Hoseini M, Silman A (2007). Relationship between change in skin score and disease outcome in diffuse cutaneous systemic sclerosis: application of a latent linear trajectory model.. Arthritis Rheum.

[15] Steen VD, Medsger TA (2001). Improvement in skin thickening in systemic sclerosis associated with improved survival.. Arthritis Rheum.

[16] Whitfield ML, Finlay DR, Murray JI, Troyanskaya OG, Chi JT (2003). Systemic and cell type-specific gene expression patterns in scleroderma skin.. Proc Natl Acad Sci U S A.

[17] Gardner H, Shearstone JR, Bandaru R, Crowell T, Lynes M (2006). Gene profiling of scleroderma skin reveals robust signatures of disease that are imperfectly reflected in the transcript profiles of explanted fibroblasts.. Arthritis Rheum.

[18] Eisen MB, Spellman PT, Brown PO, Botstein D (1998). Cluster analysis and display of genome-wide expression patterns.. Proc Natl Acad Sci U S A.

[19] Sakkas LI, Xu B, Artlett CM, Lu S, Jimenez SA (2002). Oligoclonal T cell expansion in the skin of patients with systemic sclerosis.. J Immunol.

[20] Kraling BM, Juhasz I, Freundlich B, Maul GG, Jimenez SA (1996). Transplantation of systemic sclerosis (SSc) skin grafts into severe combined immunodeficient mice: increased presence of SSc leukocytes in SSc skin grafts and autoantibody production following autologous leukocyte transfer.. Pathobiology.

[21] Kraling BM, Maul GG, Jimenez SA (1995). Mononuclear cellular infiltrates in clinically involved skin from patients with systemic sclerosis of recent onset predominantly consist of monocytes/macrophages.. Pathobiology.

[22] Yurovsky VV, Sutton PA, Schulze DH, Wigley FM, Wise RA (1994). Expansion of selected V delta 1+ gamma delta T cells in systemic sclerosis patients.. J Immunol.

[23] Fleischmajer R, Perlish JS, Reeves JR (1977). Cellular infiltrates in scleroderma skin..

[24] Trowbridge IS, Thomas ML (1994). CD45: an emerging role as a protein tyrosine phosphatase required for lymphocyte activation and development.. Annu Rev Immunol.

[25] Trowbridge IS, Ostergaard HL, Johnson P (1991). CD45: a leukocyte-specific member of the protein tyrosine phosphatase family.. Biochim Biophys Acta.

[26] Koretzky GA, Picus J, Thomas ML, Weiss A (1990). Tyrosine phosphatase CD45 is essential for coupling T-cell antigen receptor to the phosphatidyl inositol pathway.. Nature(London).

[27] Sewell WA, Brown MH, Clipstone NA, Crumpton MJ (1989). The T lymphocyte CD2 antigen–genetic and functional studies.. Transplant Proc.

[28] Sewell WA, Brown MH, Dunne J, Owen MJ, Crumpton MJ (1986). Molecular cloning of the human T-lymphocyte surface CD2 (T11) antigen.. Proc Natl Acad Sci U S A.

[29] Hale G, Xia MQ, Tighe HP, Dyer MJ, Waldmann H (1990). The CAMPATH-1 antigen (CDw52).. Tissue Antigens.

[30] Ledbetter JA, Evans RL, Lipinski M, Cunningham-Rundles C, Good RA (1981). Evolutionary conservation of surface molecules that distinguish T lymphocyte helper/inducer and cytotoxic/suppressor subpopulations in mouse and man.. J Exp Med.

[31] Sayers TJ, Lloyd AR, McVicar DW, O'Connor MD, Kelly JM (1996). Cloning and expression of a second human natural killer cell granule tryptase, HNK-Tryp-2/granzyme 3.. J Leukoc Biol.

[32] Przetak MM, Yoast S, Schmidt BF (1995). Cloning of cDNA for human granzyme 3.. FEBS Lett.

[33] Smyth MJ, Hulett MD, Thia KY, Young HA, Sayers TJ (1995). Cloning and characterization of a novel NK cell-specific serine protease gene and its functional 5′-flanking sequences.. Immunogenetics.

[34] Baker E, Sayers TJ, Sutherland GR, Smyth MJ (1994). The genes encoding NK cell granule serine proteases, human tryptase-2 (TRYP2) and human granzyme A (HFSP), both map to chromosome 5q11-q12 and define a new locus for cytotoxic lymphocyte granule tryptases.. Immunogenetics.

[35] Yoshida R, Imai T, Hieshima K, Kusuda J, Baba M (1997). Molecular cloning of a novel human CC chemokine EBI1-ligand chemokine that is a specific functional ligand for EBI1, CCR7.. J Biol Chem.

[36] Jensen PE, Weber DA, Thayer WP, Chen X, Dao CT (1999). HLA-DM and the MHC class II antigen presentation pathway.. Immunol Res.

[37] Jensen PE, Weber DA, Thayer WP, Westerman LE, Dao CT (1999). Peptide exchange in MHC molecules.. Immunol Rev.

[38] Cresswell P (1994). Assembly, transport, and function of MHC class II molecules.. Annu Rev Immunol.

[39] Gore Y, Starlets D, Maharshak N, Becker-Herman S, Kaneyuki U (2007). Macrophage migration inhibitory factor (MIF) induces B cell survival by activation of a CD74/CD44 receptor complex.. J Biol Chem.

[40] Lantner F, Starlets D, Gore Y, Flaishon L, Yamit-Hezi A (2007). CD74 induces TAp63 expression leading to B-cell survival.. Blood.

[41] Wagtmann N, Rojo S, Eichler E, Mohrenweiser H, Long EO (1997). A new human gene complex encoding the killer cell inhibitory receptors and related monocyte/macrophage receptors.. Curr Biol.

[42] Arm JP, Nwankwo C, Austen KF (1997). Molecular identification of a novel family of human Ig superfamily members that possess immunoreceptor tyrosine-based inhibition motifs and homology to the mouse gp49B1 inhibitory receptor.. J Immunol.

[43] Sulahian TH, Hogger P, Wahner AE, Wardwell K, Goulding NJ (2000). Human monocytes express CD163, which is upregulated by IL-10 and identical to p155.. Cytokine.

[44] LeClair RJ, Durmus T, Wang Q, Pyagay P, Terzic A (2007). Cthrc1 is a novel inhibitor of transforming growth factor-beta signaling and neointimal lesion formation.. Circ Res.

[45] Pyagay P, Heroult M, Wang Q, Lehnert W, Belden J (2005). Collagen triple helix repeat containing 1, a novel secreted protein in injured and diseased arteries, inhibits collagen expression and promotes cell migration.. Circ Res.

[46] Whitfield ML, George LK, Grant GD, Perou CM (2006). Common markers of proliferation.. Nat Rev Cancer.

[47] Perou CM, Sorlie T, Eisen MB, van de RM, Jeffrey SS (2000). Molecular portraits of human breast tumours.. Nature(London).

[48] Perou CM, Jeffrey SS, van de RM, Rees CA, Eisen MB (1999). Distinctive gene expression patterns in human mammary epithelial cells and breast cancers.. Proc Natl Acad Sci U S A.

[49] Whitfield ML, Sherlock G, Saldanha AJ, Murray JI, Ball CA (2002). Identification of genes periodically expressed in the human cell cycle and their expression in tumors.. Mol Biol Cell.

[50] Fleischmajer R, Perlish JS (1977). [3H]Thymidine labeling of dermal endothelial cells in scleroderma.. J Invest Dermatol.

[51] Kazandjian S, Fiessinger JN, Camilleri JP, Dadoune JP, Housset E (1982). Endothelial cell renewal in skin of patients with progressive systemic sclerosis (PSS): an in vitro autoradiographic study.. Acta Derm Venereol.

[52] Rajkumar VS, Howell K, Csiszar K, Denton CP, Black CM (2005). Shared expression of phenotypic markers in systemic sclerosis indicates a convergence of pericytes and fibroblasts to a myofibroblast lineage in fibrosis.. Arthritis Res Ther.

[53] Douglas SA, Sulpizio AC, Piercy V, Sarau HM, Ames RS (2000). Differential vasoconstrictor activity of human urotensin-II in vascular tissue isolated from the rat, mouse, dog, pig, marmoset and cynomolgus monkey.. Br J Pharmacol.

[54] Ames RS, Sarau HM, Chambers JK, Willette RN, Aiyar NV (1999). Human urotensin-II is a potent vasoconstrictor and agonist for the orphan receptor GPR14.. Nature.

[55] Grieco P, Carotenuto A, Campiglia P, Marinelli L, Lama T (2005). Urotensin-II receptor ligands. From agonist to antagonist activity.. J Med Chem.

[56] Sorlie T, Perou CM, Tibshirani R, Aas T, Geisler S (2001). Gene expression patterns of breast carcinomas distinguish tumor subclasses with clinical implications.. Proc Natl Acad Sci U S A.

[57] Liu Y, Hayes DN, Nobel A, Marron JS (2007). Statistical Significance of Clustering for High Dimension Low Sample Size Data.. Journal of the American Statistical Association Submitted.

[58] Monti S, Tamayo P, Mesirov JP, Golub TR (2003). Consensus Clustering: A resampling-based method for class discovery and visualization of gene expression microarray data.. Machine Learning.

[59] Raychaudhuri S, Stuart JM, Altman RB (2000). Principal components analysis to summarize microarray experiments: application to sporulation time series.. Pac Symp Biocomput.

[60] Margolin AA, Greshock J, Naylor TL, Mosse Y, Maris JM (2005). CGHAnalyzer: a stand-alone software package for cancer genome analysis using array-based DNA copy number data.. Bioinformatics.

[61] Segal E, Friedman N, Koller D, Regev A (2004). A module map showing conditional activity of expression modules in cancer.. Nat Genet.

[62] Stuart JM, segal E, Koller D, Kim SK (2003). A Gene-Coexpression Network for Global Discovery of Conserved Genetic Modules..

[63] Ashburner M, Ball CA, Blake JA, Botstein D, Butler H (2000). Gene ontology: tool for the unification of biology. The Gene Ontology Consortium..

[64] Palmer C, Diehn M, Alizadeh AA, Brown PO (2006). Cell-type specific gene expression profiles of leukocytes in human peripheral blood.. BMC Genomics.

[65] Funk CD (2001). Prostaglandins and leukotrienes: advances in eicosanoid biology.. Science.

[66] Crofford LJ, Wilder RL, Ristimaki AP, Sano H, Remmers EF (1994). Cyclooxygenase-1 and -2 expression in rheumatoid synovial tissues. Effects of interleukin-1 beta, phorbol ester, and corticosteroids.. J Clin Invest.

[67] Kojima F, Naraba H, Sasaki Y, Beppu M, Aoki H (2003). Prostaglandin E2 is an enhancer of interleukin-1beta-induced expression of membrane-associated prostaglandin E synthase in rheumatoid synovial fibroblasts.. Arthritis Rheum.

[68] Westman M, Korotkova M, af Klint E, Stark A, Audoly LP (2004). Expression of microsomal prostaglandin E synthase 1 in rheumatoid arthritis synovium.. Arthritis Rheum.

[69] Trebino CE, Stock JL, Gibbons CP, Naiman BM, Wachtmann TS (2003). Impaired inflammatory and pain responses in mice lacking an inducible prostaglandin E synthase.. Proc Natl Acad Sci U S A.

[70] Sheibanie AF, Khayrullina T, Safadi FF, Ganea D (2007). Prostaglandin E2 exacerbates collagen-induced arthritis in mice through the inflammatory interleukin-23/interleukin-17 axis.. Arthritis Rheum.

[71] Ross DT, Scherf U, Eisen MB, Perou CM, Rees C (2000). Systematic variation in gene expression patterns in human cancer cell lines.. nat Genet.

[72] Tusher VG, Tibshirani R, Chu G (2001). Significance analysis of microarrays applied to the ionizing radiation response.. Proc Natl Acad Sci U S A.

[73] Alizadeh AA, Eisen MB, Davis RE, Ma C, Lossos IS (2000). Distinct Types of Diffuse Large B-Cell Lymphoma Identified by Gene Expression Profiling.. Nature(London).

[74] Sorlie T, Tibshirani R, Parker J, Hastie T, Marron JS (2003). Repeated observation of breast tumor subtypes in independent gene expression data sets.. Proc Natl Acad Sci U S A.

[75] Garber ME, Troyanskaya OG, Schluens K, Petersen S, Thaesler Z (2001). Diversity of gene expression in adenocarcinoma of the lung.. Proc Natl Acad Sci U S A.

[76] Segal E, Sirlin CB, Ooi C, Adler AS, Gollub J (2007). Decoding global gene expression programs in liver cancer by noninvasive imaging.. Nat Biotechnol.

[77] Diehn M, Nardini C, Wang DS, McGovern S, Jayaraman M (2008). Identification of noninvasive imaging surrogates for brain tumor gene-expression modules.. Proc Natl Acad Sci U S A.

[78] Meighan-Mantha RL, Hsu DK, Guo Y, Brown SA, Feng SL (1999). The mitogen-inducible Fn14 gene encodes a type I transmembrane protein that modulates fibroblast adhesion and migration.. J Biol Chem.

[79] Wiley SR, Cassiano L, Lofton T, Davis-Smith T, Winkles JA (2001). A novel TNF receptor family member binds TWEAK and is implicated in angiogenesis.. Immunity.

[80] Chicheportiche Y, Chicheportiche R, Sizing I, Thompson J, Benjamin CB (2002). Proinflammatory activity of TWEAK on human dermal fibroblasts and synoviocytes: blocking and enhancing effects of anti-TWEAK monoclonal antibodies.. Arthritis Res.

[81] Novoradovskaya N, Whitfield ML, Basehore LS, Novoradovsky A, Pesich R (2004). Universal Reference RNA as a standard for microarray experiments.. BMC Genomics.

[82] Saldanha AJ (2004). Java Treeview–extensible visualization of microarray data.. Bioinformatics.

[83] Reich M, Liefeld T, Gould J, Lerner J, Tamayo P (2006). GenePattern 2.0.. Nat Genet.

[84] Pruitt KD, Maglott DR (2001). RefSeq and LocusLink: NCBI gene-centered resources.. Nucleic Acids Research.

[85] Heid CA, Stevens J, Livak KJ, Williams PM (1996). Real time quantitative PCR..

[86] Livak KJ, Schmittgen TD (2001). Analysis of relative gene expression data using real-time quantitative PCR and the 2(-Delta Delta C(T)) Method.. Methods.

