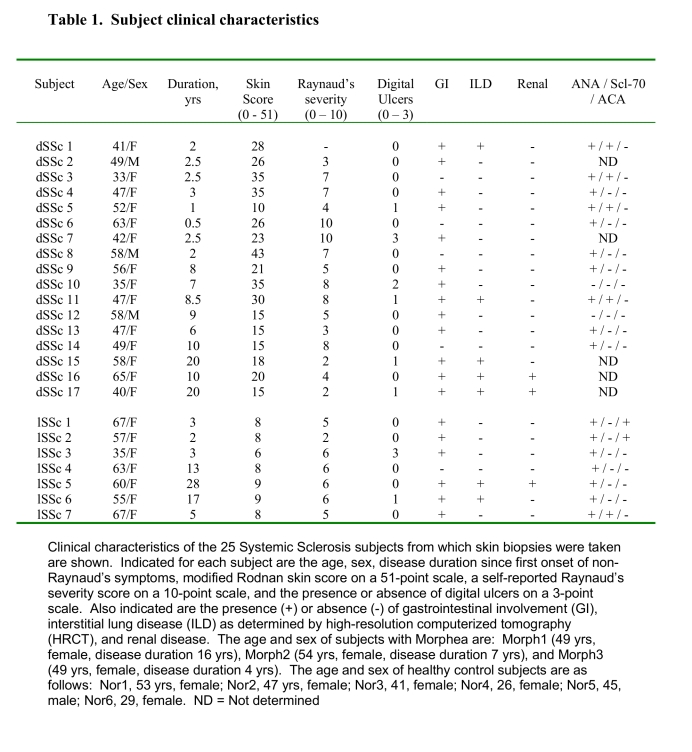# Correction: Molecular Subsets in the Gene Expression Signatures of Scleroderma Skin

**DOI:** 10.1371/annotation/05bed72c-c6f6-4685-a732-02c78e5f66c2

**Published:** 2008-10-08

**Authors:** Ausra Milano, Sarah A. Pendergrass, Jennifer L. Sargent, Lacy K. George, Timothy H. McCalmont, M. Kari Connolly, Michael L. Whitfield

In the eighth column of Table 1, five patients were incorrectly listed as having ILD. Please view the corrected table here:

**Figure:**