# Supplementary figures and images for: Molecular Subsets in the Gene Expression Signatures of Scleroderma Skin
Source: PLoS One. 2008 Jul 16;3(7):e2696. doi: 10.1371/journal.pone.0002696 (PMC2481301; doi:10.1371/journal.pone.0002696)

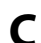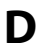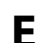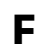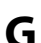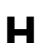[illegible]

Supplement: Figure S1 — Gene expression signatures in scleroderma. 4,149 probes that changed at least 2-fold from their median value on at least two microarrays were selected from 75 microarray hybridizations representing 61 biopsies. Probes and microarrays were ordered by 2-dimensional average linkage hierarchical clustering. This clustering shows that the dSSc, lSSc, morphea samples form distinct groups largely stratified by their clinical diagnosis. A. The unsupervised hierarchical clustering dendrogram shows the relationship among the samples using this list of 4,149 probes. Samples names have been color-coded by their clinical diagnosis: dSSc in red, lSSc in orange, morphea and EF in black, and healthy controls (Nor) in green. Forearm (FA) and Back (B) are indicated for each sample. Solid arrows indicate the 14 of 22 forearm-back pairs that cluster next to one another; dashed arrows indicate the additional 3 forearm-back pairs that cluster with only a single sample between them. Technical replicates are indicated by the labels (a), (b) or (c). 9 out of 14 technical replicates cluster immediately beside one another. B. Overview of the gene expression profiles for the 4,149 probes. Each probe has been centered on its median expression value across all samples analyzed. Measurements that are above the median are colored red and those below the median are colored green. The intensity of the color is directly proportional to the fold change. Groups of genes on the right hand side indicated with colored bars are shown in greater detail in panels C – H. C. Immunoglobulin genes expressed highly in a subset of patients with dSSc and in patients with morphea, D. proliferation signature, E. collagen and extracelluar matrix components, F. genes typically associated with the presence of T-lymphocyes and macrophages, G. Genes showing low expression in dSSc, H. Heterogeneous expression cluster that is high in lSSc and a subset of dSSc. This figure shows all gene names associated with the panels in f [file pone.0002696.s001.pdf]

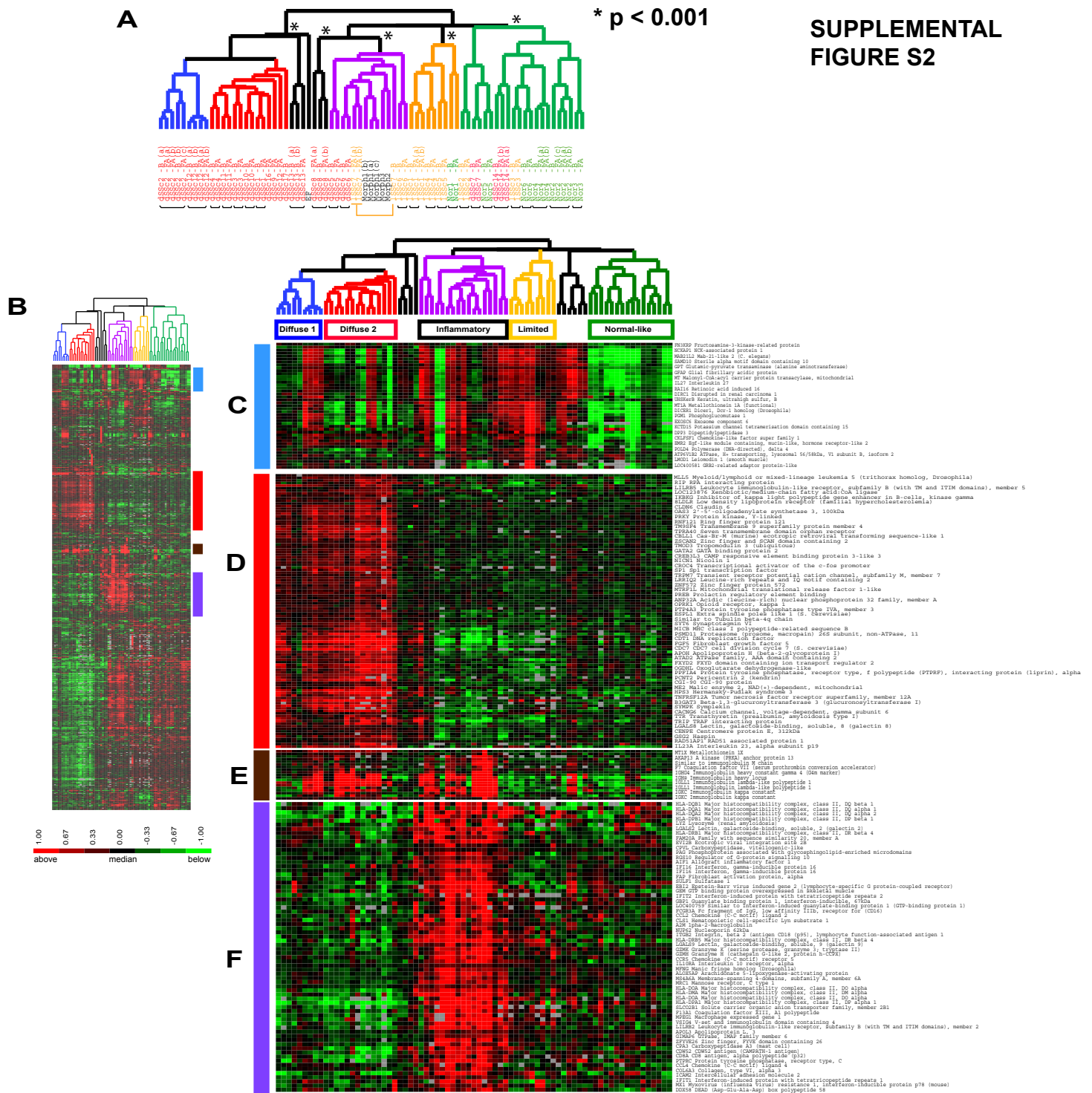

Supplement: Figure S2 — Cluster analysis using the scleroderma intrinsic gene set. The 995 most ‘intrinsic’ genes selected from 75 microarray hybridizations analyzing 34 individuals. Two major branches of the dendrogram tree are evident which divide a subset of the dSSc samples from all other samples. Within these major groups are smaller branches with identifiable biological themes, which have been colored accordingly: blue for diffuse 1, red for diffuse 2, purple for inflammatory, orange for limited and green for normal-like. Statistically significant clusters (p<0.001) identified by SigClust are indicated by an asterisk (*) at the lowest significant branch. A. Experimental sample hierarchical clustering dendrogram. Black bars indicate forearm-back pairs which cluster together based on this analysis. B. Scaled down overview of the intrinsic gene expression signatures. C. Limited SSc gene expression -cluster. D. Proliferation cluster. E. Immunoglobulin gene expression cluster. F. T-lymphocyte and IFNγ gene expression cluster. This file shows all gene names associated with the panels in figure 2 and is designed to be viewed in a digital format only so that one can zoom in to read the gene names. (6.69 MB PDF) [file pone.0002696.s002.pdf]

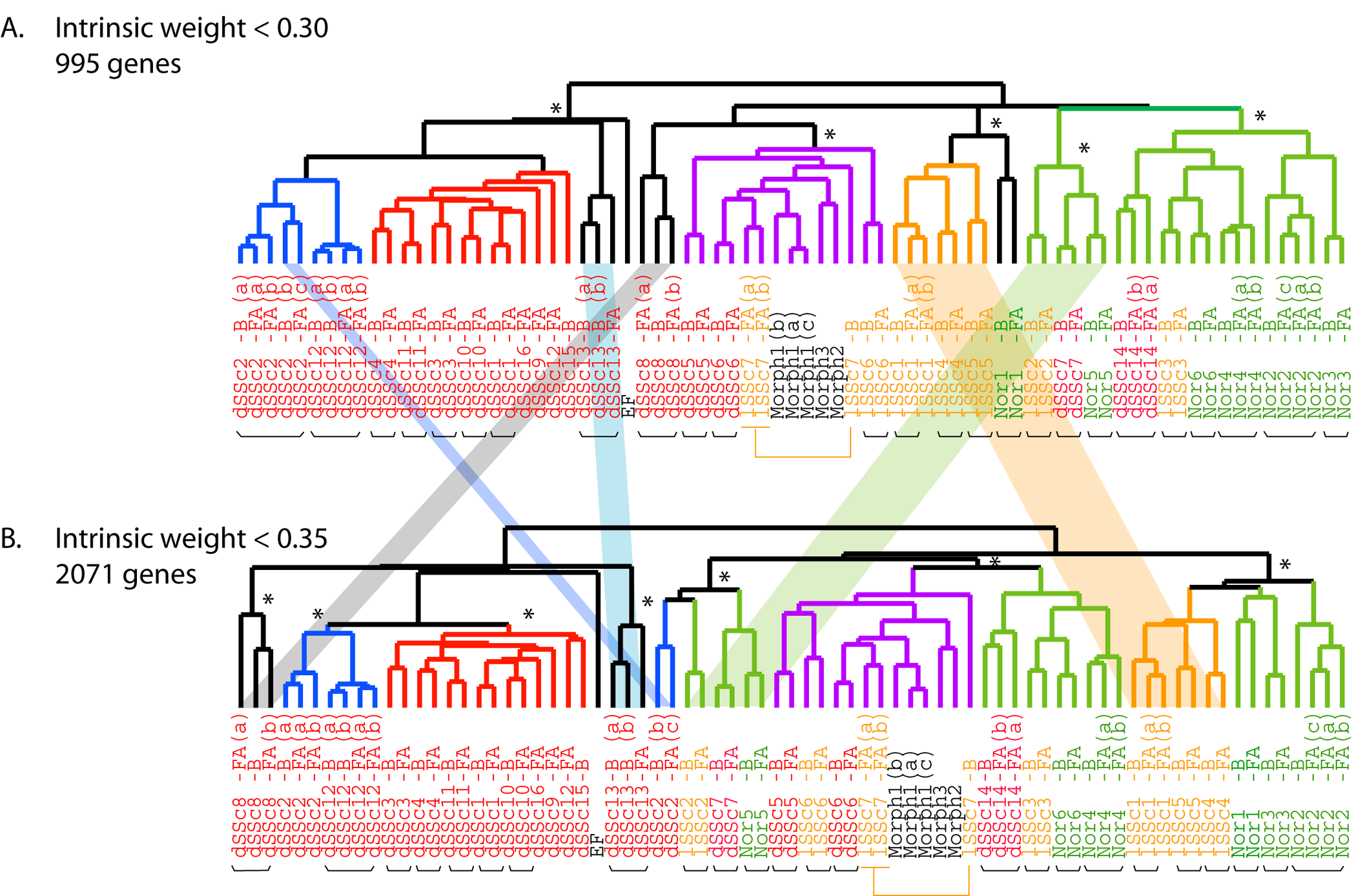

Supplement: Figure S3 — Robustness of intrinsic clustering. Hierarchical clustering was performed with two different sets of intrinsic genes. A. 995 intrinsic genes (weight <0.3; 4% FDR), B. 2071 intrinsic genes (weight <0.35, 5% FDR). Statistically significant clusters (p<0.05) as determined by SigClust are indicated by an asterisk (*). Transparent bars indicate the movement of groups of samples. The major clusters are recapitulated with this larger set of genes. (1.07 MB TIF) [file pone.0002696.s003.tif]

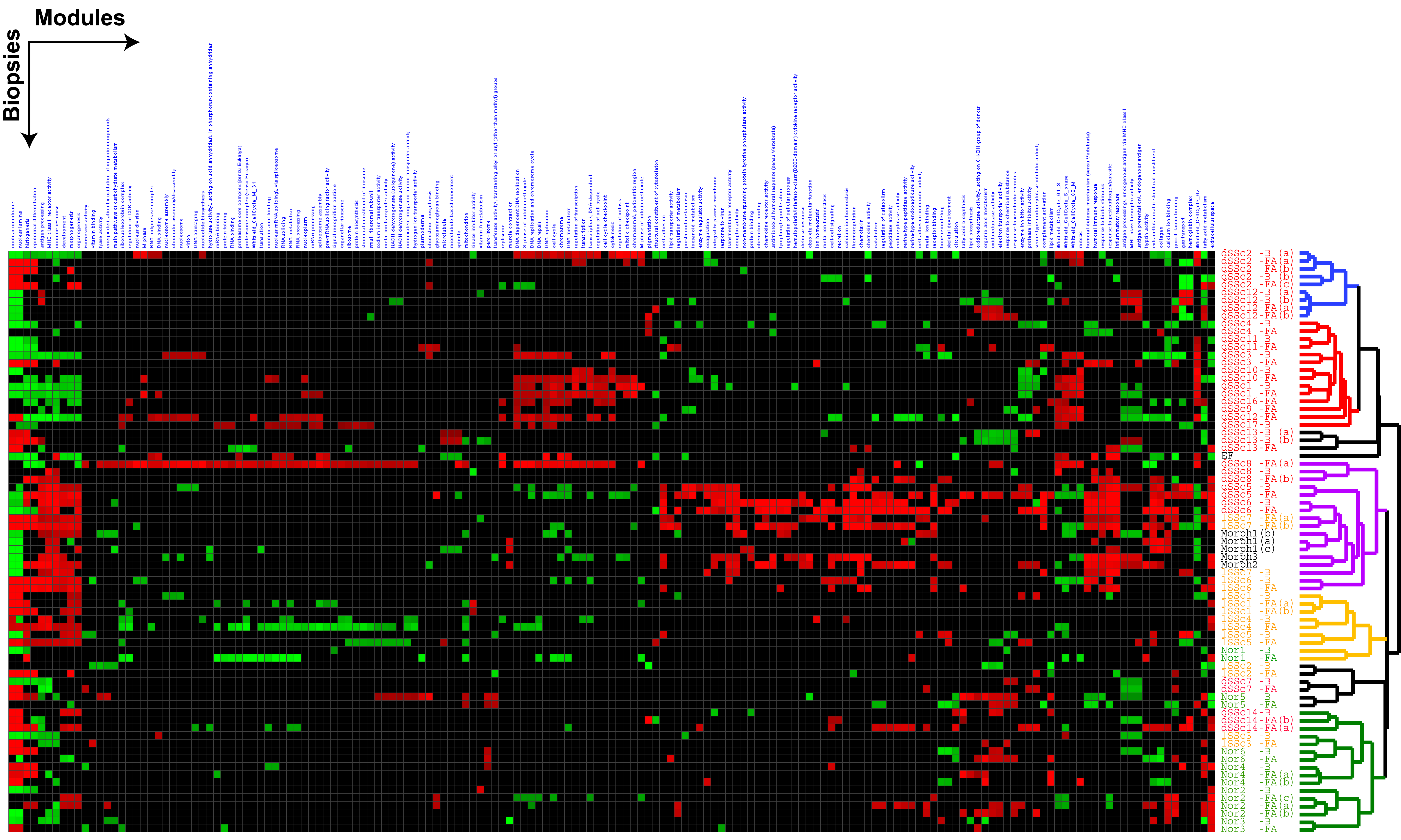

Supplement: Figure S4 — Scleroderma Module Map. Module map of the Gene Ontology (GO) Biological Processes differentially expressed among the scleroderma samples is shown. Each column represents a single microarray and each row represents a single GO Biological process. Patient samples are organized as described in Figure 2. Only modules that were significantly enriched (minimum 2-fold change, p<0.05) on at least 4 micoarrays are shown. The average expression of the gene hits from each enriched gene set is displayed here. Only gene sets that show significant differences after multiple hypothesis testing were included. This figure is best viewed in PDF format in order to read all modules names. (2.41 MB TIF) [file pone.0002696.s004.tif]

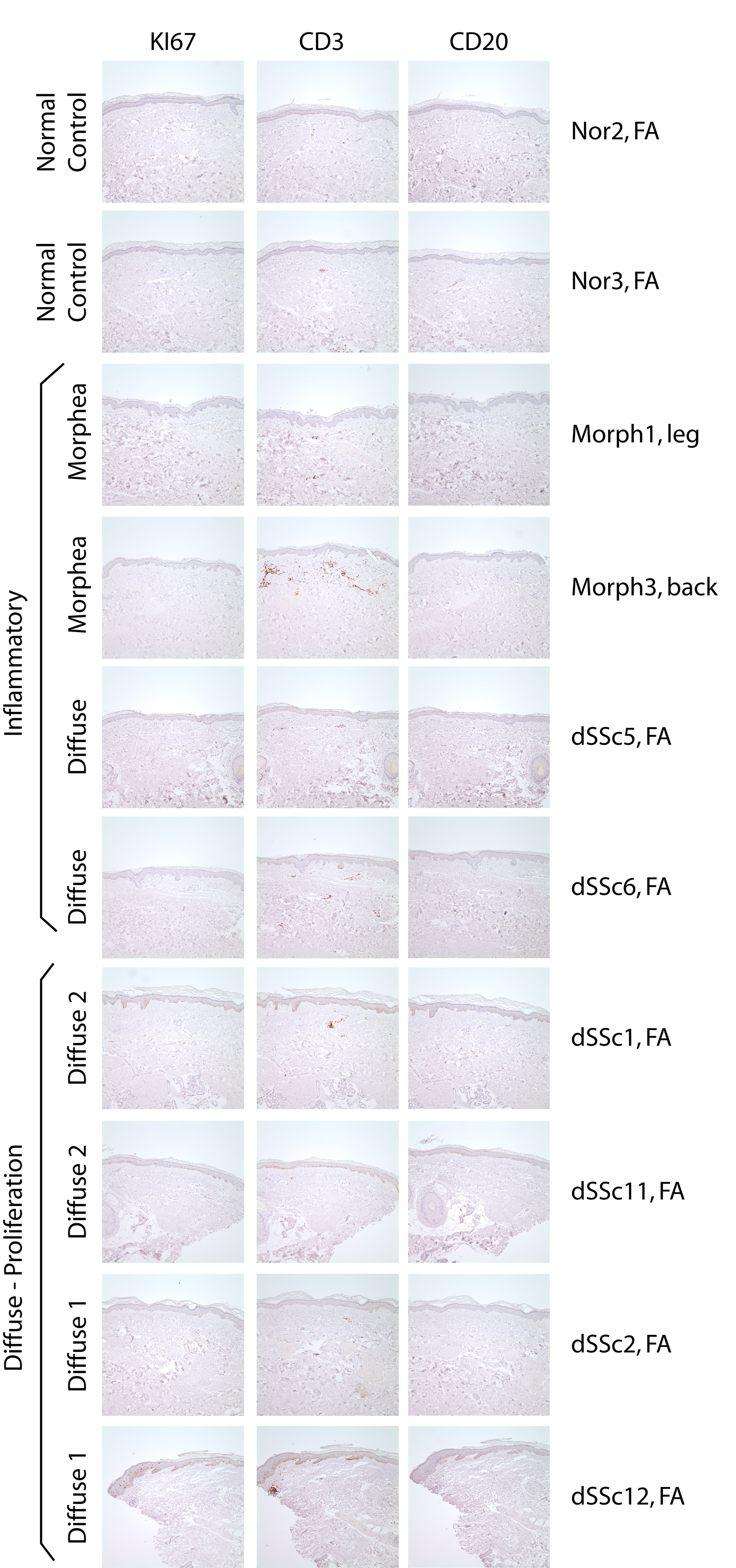

Supplement: Figure S5 — Immunohistochemistry for lymphocyte subsets and proliferating cells in scleroderma skin. Lymphocyte subsets in forearm biopsies of six dSSc patients, the leg and back specimens of two morphea patient and forearm samples of two healthy control were analyzed by immunohistochemistry. Paraffin sections were stained for T cells (CD3), B cells (CD20) and proliferating cells (KI67). (Magnification: ×200). See table 4 for detailed quantification. (9.60 MB TIF) [file pone.0002696.s005.tif]
